# Advanced Multiscale Modeling for Revealing Anomalous Fluid Transport Induced by Confinement Interfacial Layer Reconstruction in Sub‐10 nm Space

**DOI:** 10.1002/advs.75496

**Published:** 2026-04-29

**Authors:** Xiang Zhang, Bing Wei, Jingyi Wang, Runnan Wu

**Affiliations:** ^1^ State Key Laboratory of Oil and Gas Reservoir Geology and Exploitation Southwest Petroleum University Chengdu China; ^2^ Department of Energy and Power Engineering Tsinghua University Beijing China; ^3^ Department of Building Environment and Energy Engineering The Hong Kong Polytechnic University Hongkong China; ^4^ College of Chemistry and Chemical Engineering Southwest Petroleum University Chengdu China

**Keywords:** apparent viscosity, interfacial layer model, intermolecular interactions, molecular dynamics, nanoconfined space

## Abstract

Fluid transportation in nanoconfined space is crucial for comprehensively understanding the distinctive flows in nano‐porous media, particularly in shale and tight reservoirs. Nanoscale confinement induces substantial deviations in fluid apparent viscosity from the bulk state. These discrepancies are significantly influenced by interfacial layer effects, which have been widely recognized as the dominant contributors to viscosity amplification. However, existing theoretical models fail to adequately quantify the interfacial layer characteristics for confined fluids with dimensions less than 100 nm. By integrating the insights from experimental studies and molecular dynamics simulations, we developed an advanced mathematical model to characterize the interfacial properties of fluids confined in channels below 10 nm. Upon incorporating specific channel material properties, temperature conditions, and fluid characteristics into this model, it enables characterization of both the interfacial layer thickness and viscosity. Notably, the molecular interaction coefficient in this model demonstrates a strong linear correlation with alkane chain length, showing minimal dependence on channel dimensions but high sensitivity to temperature. The interfacial layer exhibits a 6.89‐fold enhancement in viscosity compared to the bulk state at the channel height of 70 nm, establishing it as the predominant determinant of the anomalous transportation of nanoconfined fluid.

## Graphical abstract

Integrating experimental insights with molecular dynamics simulations, we establish an advanced mathematical model for fluids confined in sub‐10‐nm channels, enabling quantitative prediction of interfacial layer thickness and viscosity by accounting for channel material, temperature, and fluid properties.

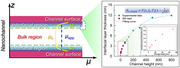

## 1 Introduction

Nanoconfinement amplifies the contribution of solid/fluid interface properties, e.g., interfacial tension, wettability, adsorption rate, etc., to fluid flow behavior. Consequently, fluid properties (viscosity, diffusion coefficient, and evaporation fluxes, etc.) in nanoconfined space exhibit substantial differences from those in the bulk state, and their apparent values describe the influence of nanoconfinement [1, 2, 3, 4, 5, 6, 7, 8]. The apparent viscosity behavior of nanoconfined fluids plays a vital role in the hydrocarbon transportation during the development of unconventional reservoirs, characterized by a significant presence of nanosized pores [9, 10, 11, 12, 13]. Furthermore, it has spurred a myriad of investigations across diverse domains, including nanoconfined catalysis, rectification, and electrochemistry [14, 15, 16, 17, 18, 19].

The exploration of fluid apparent viscosity has drawn an increasingly attention with advancements in nanofabrication and miniaturized analysis technologies [20, 21, 22, 23, 24, 25]. Certain researchers have demonstrated that the apparent viscosity of nanoconfined fluids surpassed that in bulk. For instance, Israelachvili [26] conducted experimental research on the apparent viscosity of nanoconfined fluids (tetradecane, water, and saline solution) by using the surface forces apparatus (SFA). The results indicated that the fluid apparent viscosity was 10% higher than that in the bulk state. To apply this method, the amplitudes and frequencies should be low to generate low shear rates, where inertial and acceleration terms could be ignored, and the only resistance to flow was due to fluid viscosity. Li et al. [27] improved the accuracy of apparent viscosity measurement by the pressurized capillary flow method. It was found that the water viscosity was 2.5 times higher than that in the bulk state in a space size of 200 nm. Similar observations were reported by Mawatari et al. [2], Kazoe et al. [28], Tas et al. [29], and Haneveld et al. [30]. The size effect intensifies fluid‐wall molecular interactions as confinement dimension transitions from microscale to nanoscale. This leads to the formation of an interfacial layer, which can be understood as a near‐surface region of finite thickness where the fluid structure and transport properties deviate from those in the bulk due to surface‐induced effects [31]. The interfacial layer is considered a primary factor contributing to the increased viscosity of nanoconfined fluids relative to their bulk state [32, 33]. For example, Nazari et al. [34] studied the fluid viscosity in the interfacial layer by molecular dynamics (MD) technology. The results demonstrated that the viscosity of isopropanol and ethanol in the interfacial layer was significantly higher than their bulk values. Extensive research has indicated that the anomalous structural arrangements of fluid molecules within the interfacial layer induce a significant enhancement in flow resistance. For instance, Vo et al. [35] presented an approach for predicting nanoscale capillary imbibition via MD simulations. They claimed that the liquid layering in the vicinity of a solid surface induced a higher viscosity than that of the bulk state, leading to a slower MD uptake of fluid into the capillaries than theoretically predicted. Essentially consistent conclusions were derived in the study of Lu et al. [36]. They investigated the capillary imbibition of shale oil in nanochannels via a nanofluidic device. The authors speculated that shale oil formed an interfacial layer of two to three layers of molecules on the channel walls, which greatly increased flow resistance in the nanochannels. To thoroughly explore these potential specific structures within the interfacial layer. Kitamori et al. [37, 38] employed NMR spectroscopy results to characterize the molecular structure and dynamics of water in nanochannels. The results suggested that there existed a proton‐transfer phase that consisted of loosely coupled water molecules located within approximately 50 nm from the channel surface to decelerate the fluid flow. Precise quantification of the fluid interfacial layer thickness provides crucial insights into identifying unique flow behaviors. Based on the experimental research findings, Li et al. [27]. proposed a model for calculating the characteristic parameters of confined fluid in the interfacial layer. However, it was found that the interfacial layer thickness computed by this model might be overestimated. The empirical formula method provides an additional effective approach to examining fluid transport characteristics in the interfacial layer. For example, Zhang et al. [39] proposed an empirical formula to calculate the interfacial layer thickness of confined fluids applicable to specific conditions. It was noteworthy that the interfacial layer thickness obtained using this formula showed minimal dependence on channel height, and it did not account for temperature variations. These pioneering works have collectively confirmed that the anomalous apparent viscosity of nanoconfined fluids is closely associated with the interfacial layer effect. Despite advances in the influence of the interfacial layer on fluid transport through molecular simulations and theoretical modeling, direct experimental validation of their impact on fluid apparent viscosity remains conspicuously underdeveloped.

This work focuses on the apparent viscosity of fluid transportation in nanoconfined space by using nanofluidic and MD technology. Although considerable attention has been devoted to this field, the quantitative relationship between interfacial layer characteristics and apparent viscosity remains unclear. Moreover, the underlying mechanisms by which the interfacial layer affects fluid apparent viscosity at the microscopic scale, including channel surface roughness and molecular structure, remain largely unexplored. To address these issues, herein we present an experimental and simulated study of fluid transportation in nanoconfined space ranging from 3 to 1000 nm. The investigation of apparent viscosity of fluids, including four types of n‐alkanes with different carbon chain numbers and deionized water was first conducted via a nanofluidic device. Further analysis of fluid properties in the interfacial layer was carried out to reveal the essential mechanisms of the anomalous apparent viscosity. A novel interfacial layer model was developed by synergistically integrating experimental characterization with MD simulations. This systematic framework enables the prediction of both interfacial layer thickness and viscosity of nanoconfined fluid once the fluid type, channel surface characteristics, and operating temperature are defined. The results of this study can substantially enrich the theoretical framework of fluid transport in nanoconfined space and provide novel insights into the intricate transport mechanisms of multiphase flow under nanoconfinement conditions.

## 2 Results

### 2.1 Apparent Viscosity of Nanoconfined Fluid

**FIGURE 1:**
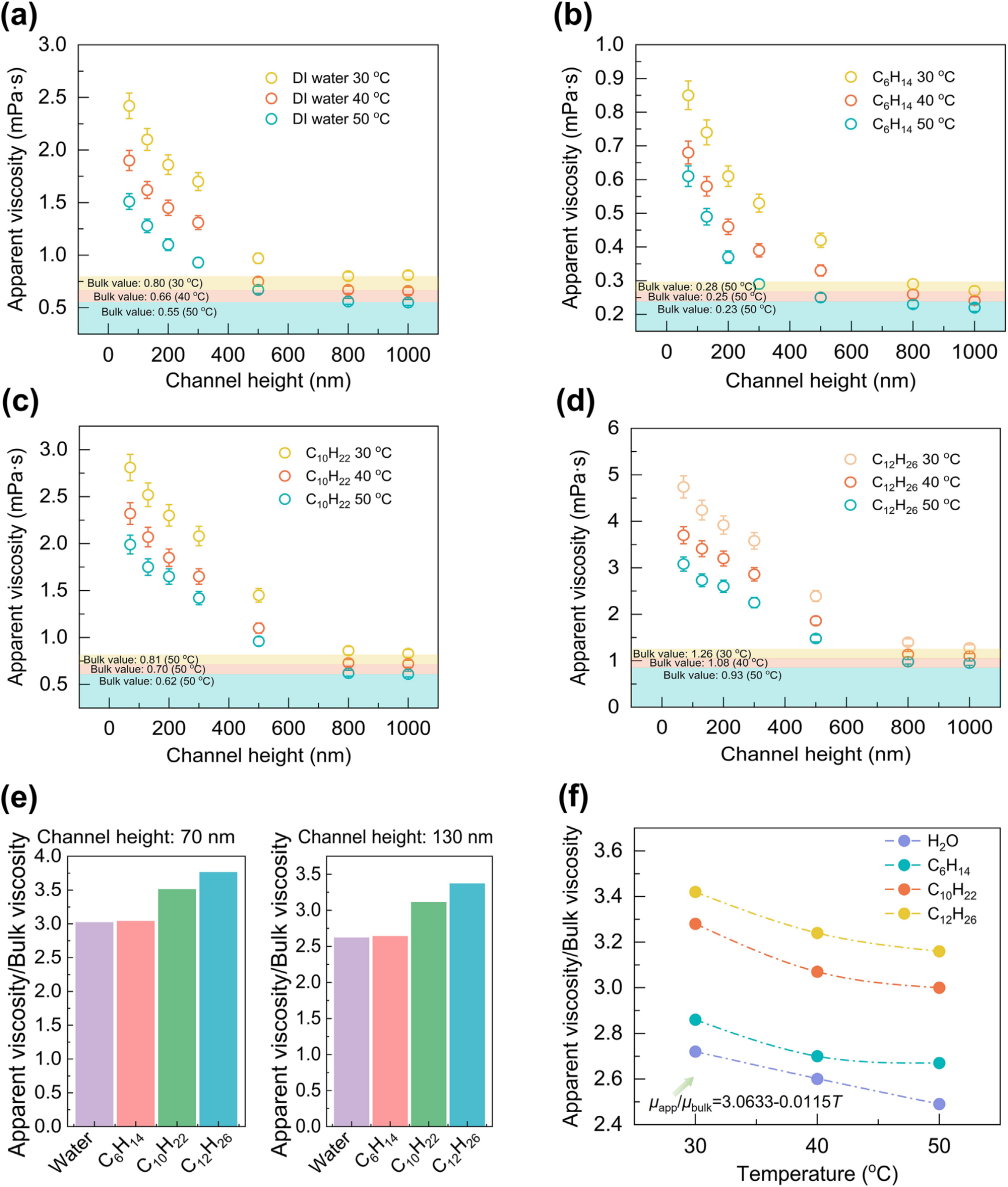
The influence of spatial scale and temperature on the apparent viscosity of nanoconfined fluids, including (a) DI water, (b) C_6_H_14_, (c) C_10_H_22_, (d) C_12_H_26_. (e) The apparent viscosity vs. bulk value with the channel height of 70 and 130 nm at 30°C. (f) The apparent viscosity vs. bulk value with the temperature from 30°C to 50°C at 130 nm.

The apparent viscosity of nanoconfined fluids, including DI water, C_6_H_14_, C_10_H_22_, and C_12_H_24_ under varying temperature and spatial scales was calculated by Equation (1) (Detailed derivation is presented in Section S3), as illustrated in Figure 1a–d. The observed trend revealed a rapid increase in fluid apparent viscosity with diminishing channel height, gradually converging to the bulk value as the channel height approached 1000 nm. As depicted in Figure 1e, the ratios of the apparent viscosity to the bulk value of fluids were 3.2, 3.25, 3.69, and 3.88 at the channel height of 70 nm. Figure 1f shows the variation in the ratios of apparent viscosity to bulk value of fluids with temperature at 130 nm. Notably, a robust linear relationship (*R* ^2^ = 0.99) was discerned between the ratio of water apparent viscosity to bulk value and temperature, whereas alkanes did not exhibit a similar linear correlation.

(1) $$\frac{\Delta x^{2}}{\Delta t}=\frac{16h^{2}\left[1-\frac{2h}{\pi w}\tanh\left(\frac{\pi w}{2h}\right)\right]}{\pi^{4}\mu }\left(\frac{4\gamma \cos\theta }{D_{h}}+P_{ex}\right)$$

where *h* and *w* stand for the height and width of the nanochannel, *µ* is the fluid apparent viscosity, *γ* is the interfacial tension between two phases, *θ* is the contact angle, *D_h_* stands for the hydraulic radius of the nanochannel, and *P_ex_* denotes the external pressure.

## 3 Discussion

### 3.1 Interfacial Layer Effect

**FIGURE 2:**
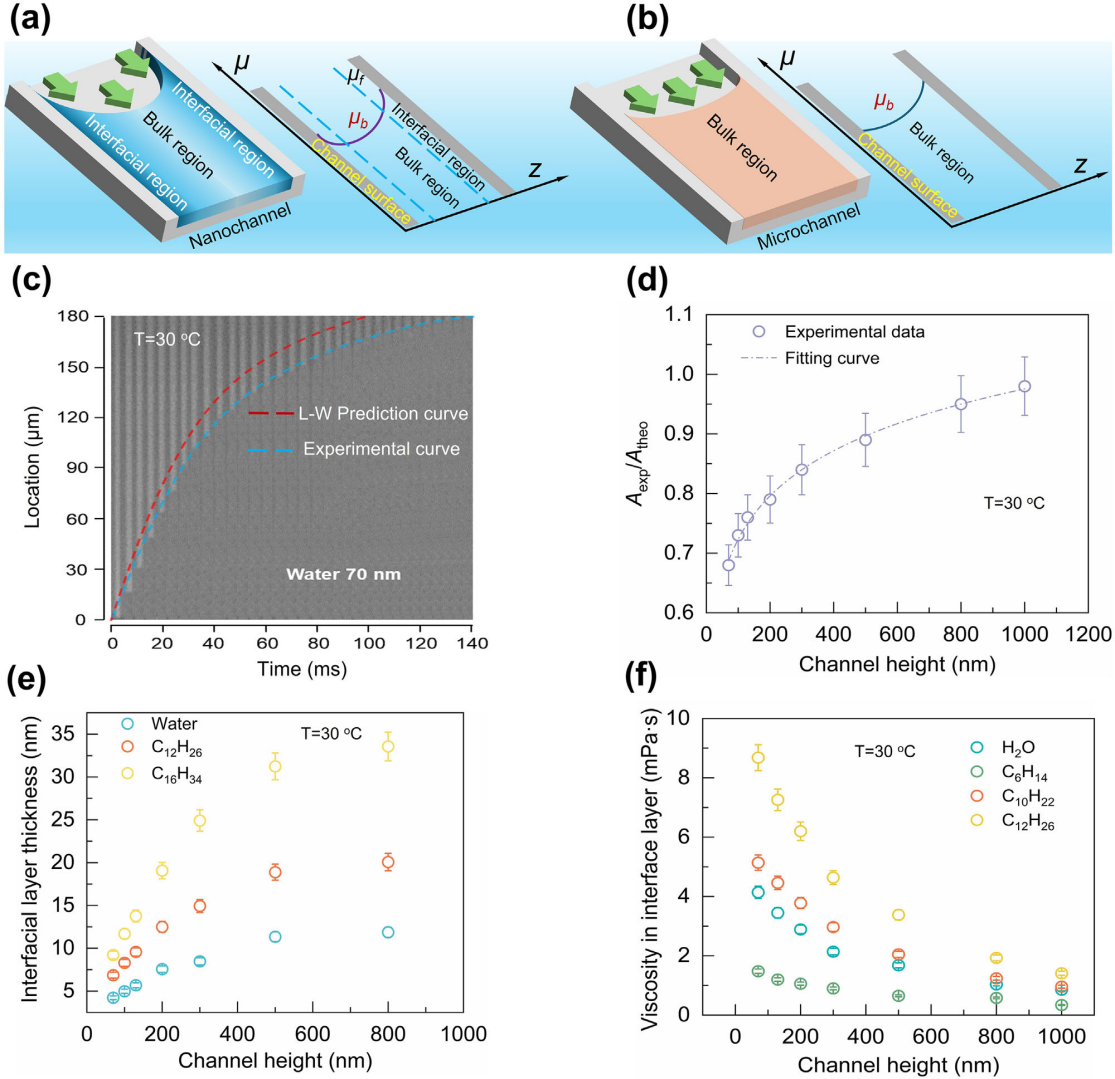
The effect of the interfacial layer on the apparent viscosity of nanoconfined fluids. The flow region and viscosity field of fluid in (a) a nanochannel and (b) a microchannel. (c) Comparison of the relationship between meniscus displacement Δ*x* and time Δ*t* during water imbibition in nanochannels (depth: 70 nm, width: 5 µm) and the predicted curve of the L‐W model. (d) The ratio of *A*_exp_ to *A*_theo_ with the channel height in water imbibition. (e) The relationship between interfacial layer thickness of different fluids (water, C_12_H_26_, and C_16_H_34_) and channel height. (f) The apparent viscosity of nanoconfined fluids in the interfacial layer.

The experimental investigation on the transportation of nanoconfined fluids has revealed that the apparent viscosity exhibited a strong dependence on channel height, temperature, and fluid type. The flow boundaries of fluids in nanoconfined space can be divided into a bulk flow region and an interfacial layer region (Figure 2a). The interfacial layer represents the region where fluid and channel wall interact with strong forces, whereas the bulk fluid properties remain unaffected by these interactions [40]. As depicted in Figure 2a, the apparent viscosity of nanoconfined fluid consisted of the interfacial layer viscosity (*µ_f_*) and bulk viscosity (*µ_b_*) [34, 36]. The ratio of *µ_f_*/*µ_b_* gradually increased as the channel height decreased from microscale (Figure 2b) to nanoscale, resulting in a marked increase in apparent viscosity. Numerous molecular simulations and experimental results have established that nanoconfined fluids display unique transport characteristics in the interfacial layer. However, there is still a significant gap in our understanding regarding both the precise characterization of interfacial fluid properties and the quantification of the influence of this interface on apparent viscosity.

The interfacial layer thickness of nanoconfined fluids can be calculated by assessing the offset degree of the experimental data with the L‐W prediction curve (Equation 2) (Figure 2c,d). Detailed calculation theory is provided in Section S4. This offset was attributed to differences between the apparent viscosity and bulk viscosity of the fluid, as shown in Figure 1. As evidenced by Figure 2e, a linear correlation emerged between temperature elevation and the progressive thinning of the water interfacial layer (See Section S4, Figure S4). This thermal dependence stems from the modified molecular dynamics, where elevated thermal energy diminishes activation energy barriers, consequently permitting boundary‐proximal molecules to transition from interfacial confinement zones into the bulk phase domain. Concomitantly, this molecular redistribution induced systematic energy dissipation in the interfacial regime, manifesting as contraction of layer thickness. The influence of fluid type on layer thickness was also examined (Figure 2e). The results indicated that the interfacial layer thickness of alkane was greater than that of water under identical conditions, and the layer thickness of long‐chain alkane exceeded that of short‐chain alkane. This observation agreed with the apparent viscosity trend of long‐chain alkane > short‐chain alkane > water (Figure 1a–d), suggesting that a larger interfacial layer thickness resulted in higher apparent viscosity. The MD results showed that stronger intermolecular interactions between hydrocarbon molecules and the channel surface, as well as more pronounced intramolecular forces among the hydrocarbon chains, contributed to a significant increase in the interfacial layer thickness. Earlier works suggested that long‐chain alkane molecules tended to distribute parallelly near the wall region to minimize interfacial energy [41, 42]. Figure 2e and Figure S5 also demonstrate an increase in interfacial layer thickness with channel height, while its proportion to the total depth (*h_a_/h*) continued to decrease, as shown in Figure S6. These trends indicated a diminishing contribution of the interfacial layer to apparent viscosity.

(2) $$\frac{A_{exp}}{A_{theo}}={\left(1-\frac{2h_{a}}{h}\right)}^{3}$$

where *A_exp_*/*A_theo_* can be obtained by comparing the Δ*x* ^2^∝Δ*t* curve between the experimental data and predicted data by the L‐W equation, *h_a_* and *h* were the interfacial layer thickness and channel height, respectively.

Simultaneously, the viscosity of fluids in the interfacial layer also exhibited an inverse proportionality with the channel height (Figure 2f). When the channel height was 70 nm, C_12_H_26_ displayed the interfacial viscosity enhancement to 8.86 mPa·s, marking a 6.89‐fold increase compared to the bulk value and a 1.78‐fold increase compared to the apparent value. This anomalous viscosity enhancement in the interfacial layer governs momentum transfer dynamics in nanoconfined systems through specific dissipation mechanisms that facilitate hydrodynamic boundary layer structural reconstruction. As demonstrated in prior studies [43], the fluid flux near the channel wall approached negligible levels when the fluid viscosity in the interfacial layer was notably high. This phenomenon originated from strong intermolecular interactions between the fluid and channel wall, which effectively immobilized adjacent fluid molecules. Such molecular immobilization led to a reduction in interfacial layer thickness, thus diminishing its contribution to the apparent viscosity of nanoconfined fluids. Therefore, both the decrease of *h_a_*/*h* and the viscosity of fluids in the interfacial layer could cause the reduction of apparent viscosity (See Section S5).

Interfacial slip may affect fluid transport when the characteristic length scale is sufficiently small. Fluid may exhibit no slip or even a negative slip length under strongly wetting conditions [44, 45]. Hydrodynamic force measurements on hydrophilic surfaces have reported an apparent slip length of 8–9 nm. This value is generally regarded as an upper‐bound estimate due to possible contributions from electrokinetic and other interfacial effects [46]. For non‐hydrophilic surfaces, experiments in a single graphene nanochannel have suggested a slip length of 16 nm [47]. In this study, the chip is fabricated from a hydrophilic material and possesses intrinsic surface roughness, both of which are expected to suppress interfacial slip. More importantly, the displacement difference calculated for the advancing meniscus front is on the micrometer scale, far exceeding the possible slip distance. Therefore, any dynamic slip effect is expected to be negligible and is thus omitted from the calculation of the interfacial layer parameters. A head loss is expected as fluid enters the nanochannel from the microchannel. For a nanofluidic device with a nanochannel height of 70 nm and a microchannel height of 500 µm, the capillary pressure drop of water in the nanochannel reaches the MPa scale, far exceeding that in the microchannel. By contrast, the local pressure loss associated with the transition (Δ *p*_local_ =  ζρ*v* ^2^/2), is negligible since the flow velocity in the spontaneous imbibition experiments is extremely low. In addition, the absence of abrupt geometric features in the transition region allows entrance effects to be neglected. Furthermore, when the channel depth is 70 nm, substantial discrepancies were observed between the L‐W model predictions and the experimental results for the spontaneous imbibition of both water and C_12_H_26_. By comparison, at a channel height of 1000 nm, the model predictions were in good agreement with the experimental observations (Figure S7). These findings indicated that the observed differences in apparent fluid viscosity arose primarily from the reduction in spatial scale, rather than from any change in the intrinsic viscosity of the liquids.

### 3.2 Interfacial Layer Model of Confined Fluid

**FIGURE 3:**
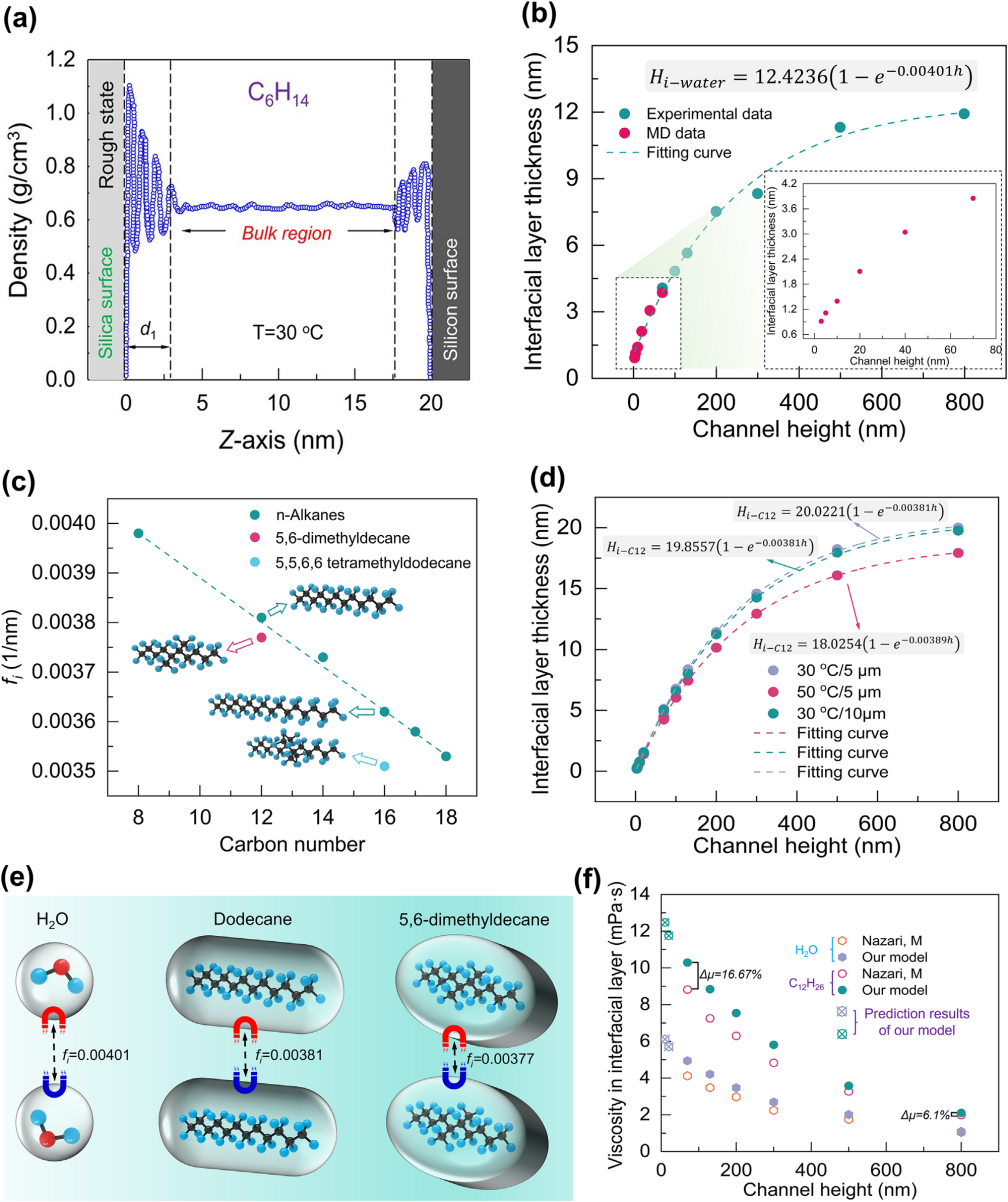
The interfacial layer model of nanoconfined fluid. (a) Density distribution of C_6_H_14_ in a nanochannel with a width of 20 nm. (b) The interfacial layer thickness model of water vs. channel height. (c) The relationship between the molecular interaction coefficient *f*_i_ and the carbon number of alkanes. (d) The influence of temperature and channel height on the interfacial layer model. (e) Schematic diagram of the influence of molecular structure on the molecular interaction coefficient. (f) Comparison of the results obtained from our model and the literature [34].

MD simulations were conducted to further investigate fluid transport behavior at the molecular level. The density distribution of liquid molecules in the interfacial layer was analyzed to elucidate the mechanisms underlying apparent viscosity observations. As shown in Figure 3a, the C_6_H_14_ density distribution within the nanochannel is divided into three distinct zones: the interfacial region where fluids interact with the silica walls, the central bulk phase, and the region adjacent to the silicon walls (Other fluids were presented in Section S6). The fluid density progressively diminished as the distance increased toward the interaction boundary, eventually reaching the levels consistent with bulk density. This oscillatory behavior near the wall is primarily attributed to the potential energy of solid‐liquid interactions, which attract more fluid molecules into the vicinity of these energy traps [48]. The density fluctuation region refers to the area characterized by strong molecular interactions. These intense interactions result in the formation of an interfacial layer, which in return leads to the deviations between the L‐W equation and experimental results. Consequently, the definition of the interfacial layer in MD studies is fundamentally aligned with that derived from experimental results. This alignment enables the fluid density fluctuation region to be treated as the interfacial layer in MD simulations. Then, the interfacial layer thickness of water within a wide spatial range from 3 to 800 nm (MD data: 3–20 nm, experimental data: 70–800 nm) was obtained as presented in Figure 3b (Other fluids were shown in Section S7). The results demonstrated that the fluid interfacial layer thickness exhibited a strong exponential relationship with channel height (*R^2^* = 0.99; Equation 3). Furthermore, at a channel height of 70 nm, the MD simulation results corresponded closely with experimental data. This agreement was characterized by a maximum error of only 4.9%, confirming the validity and reliability of our simulation study.

(3) $$H_{i-water}=12.42362\left(1-e^{-0.00401h}\right)$$

Consequently, a unified formula (Equation 4) was developed to describe the relationship between the interfacial layer thickness and channel height for different fluids based on Equations (3), as well as Equations S19–S25. This formula captures fluid specific characteristics and enables consistent predictions of interfacial layer thickness across various fluids and channel heights.

(4) $$H_{i-fluid}=h_{m}\left(1-\frac{1}{e^{f_{i}h}}\right)$$

where *H_i‐fluid_* denotes the fluid interfacial layer thickness, which gradually increases with channel height and ultimately reaches a steady state. The equilibrium thickness at this stable condition is denoted as *h_m_*. *f_i_* stands for the molecular interaction coefficient. A smaller *f_i_* value indicates a shorter effective interaction distance and stronger molecular interaction, and *h* is the channel height. The factors influencing *f_i_* were further explored, as shown in Figure 3c. The results revealed that *f_i_* exhibited a strong linear relationship with the carbon number of n‐alkanes (Equation 5, *R* ^2^ = 99%). This relationship can explain the parallel motion of alkane molecules in nanoconfined spaces [49]. In contrast, the *f_i_* of isoalkanes did not follow this linear relationship compared to n‐Alkanes (Figure 3c). This outcome was attributed to the molecular structure of isomeric alkanes giving rise to more intricate molecular motions while simultaneously modifying the effective intermolecular interaction distance, as illustrated in Figure 3e. The trend of *f_i_* for the fluids was: 5,6‐dimethyldecane < dodecane < water.

(5) $$f_{i}=0.00434-4.43923\times 10^{-5}C_{n}$$

where *C*_n_ stands for carbon number.

The effect of temperature and channel height on the interfacial layer thickness model was further examined. As shown in Figure 3d, the *h_m_* of dodecane decreased only by 0.83%, while the *f*_i_ remained constant when the channel width increased to 10 µm. However, the *h_m_* decreased by 9.97% and *f*_i_ increased by 2.1% when the temperature increased to 50°C. These results suggested that the intermolecular interaction distance was largely insensitive to channel width. The increasing temperature intensified the molecular thermal motion, resulting in a reduction of *h_m_*. On this basis, a model for fluid interfacial layer thickness can be expressed as Equation (6).

(6) $$H_{i-fluid}=F\left(s,f_{t},T\right)\left(1-\frac{1}{e^{f_{i}h}}\right)$$

In the case of the n‐alkanes, the model can be rewritten as follows:

(7) $$H_{i-fluid}=F\left(s,f_{t},T\right)\left[1-\frac{1}{e^{\left(0.00434-4.43923\times 10^{-5}C_{n}\right)h}}\right]$$

where *F*(*s*, *f*_t_, *T*) represents the functions corresponding to surface properties of materials, fluid type, and temperature. Note that Equation (2) and Equation S17 were only applicable for characterizing the fluid flow in nanochannels with heights greater than 70 nm (See Section S7, Figure S12). In contrast, the model developed in this study (Equation 7) described the fluid interfacial layer thickness in channels with heights below 20 nm. Previous studies have indicated that the Navier‐Stokes equation remains applicable at length scales of 1–2 nm [31]. Since the minimum channel size considered in this study is 3 nm, the proposed model is still within the validity range of the continuum assumption.

To verify the unique identifiability of the interfacial layer parameters derived from this model, we modified the channel wettability from hydrophilic to hydrophobic. The results indicated that the relationship between interfacial layer thickness and channel height of water no longer conformed to the trend predicted by the model (Figure S13). We further verified the influence of alkane structure. For the isomers of n‐dodecane (3,3‐dimethyldecane), the relationship between interfacial layer thickness and channel depth no longer followed the trend predicted by the model under the same experimental conditions (Figure S14). Therefore, fluid viscosity in the interfacial layer can be described as follows:

(8) $$\mu_{f}=\frac{3\kappa \left(h^{3}-8hH_{i-fluid}^{3}\right)\mu_{app}^{2}\mu_{0}}{\gamma h^{3}\cos^{2}\theta \mu_{0}-24\kappa H_{i-fluid}^{3}\mu_{app}^{2}}$$

The results derived from Equation (8) were rigorously compared with those previously reported by Li et al. [29], as illustrated in Figure 3f. Analysis revealed that the observed discrepancies progressively increased as the spatial scale decreased, culminating in a notable deviation of 16.67% at the channel height of 70 nm. Simultaneously, this model was able to predict the viscosity of nanoconfined fluids in the interfacial layer, yielding values of 11.05 mPa·s at 20 nm and 11.88 mPa·s at 10 nm. These predictions showed good agreement with the MD results (Figure S15). These findings clearly suggest that when specific material properties, temperature conditions, and fluid characteristics are incorporated into the model, it effectively characterizes both interfacial layer thickness and viscosity in nanoconfined space.

### 3.3 Microscopic Influencing Factors on Apparent Viscosity

**FIGURE 4:**
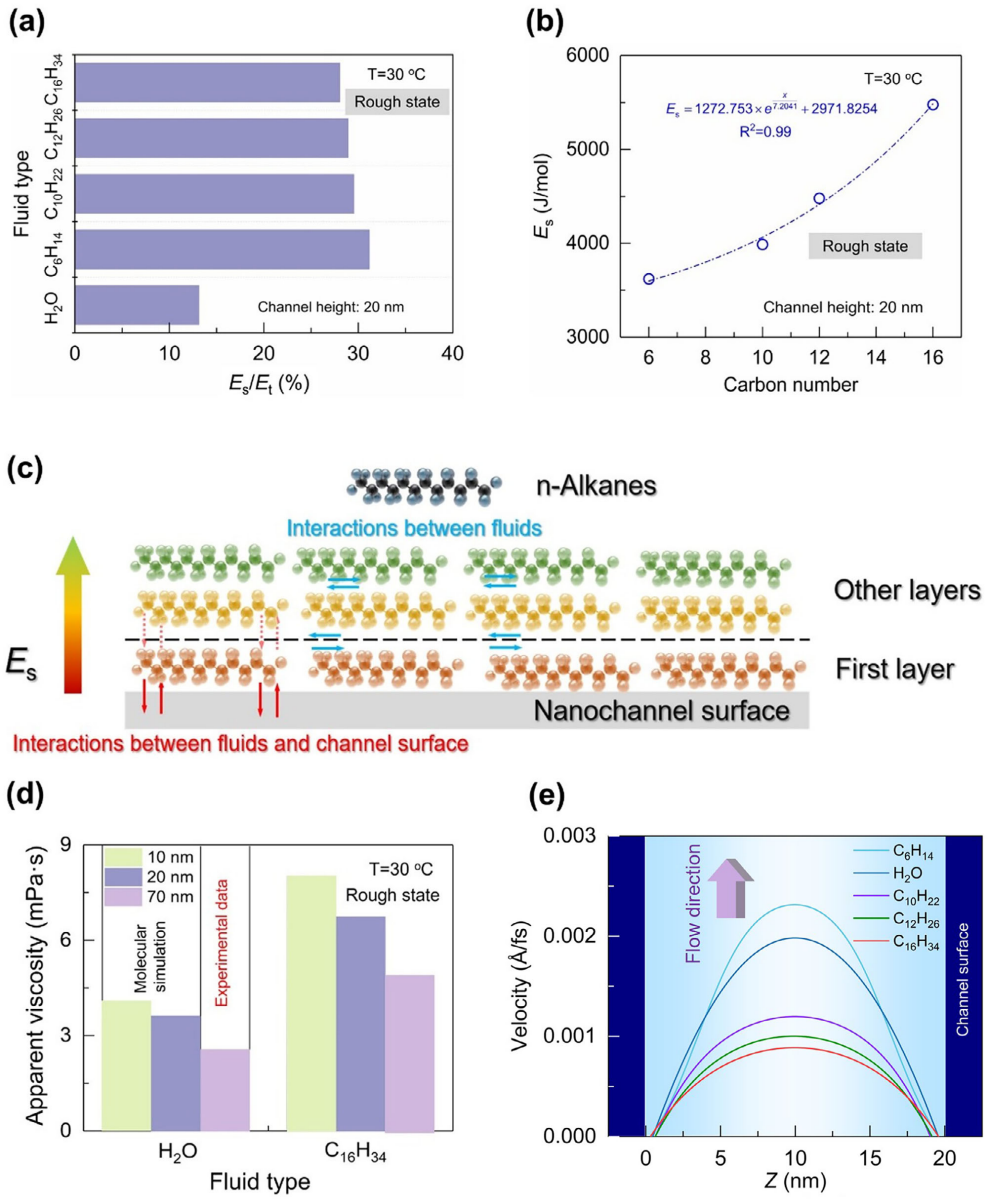
MD study on the transportation of nanoconfined fluids. (a) The proportion of the *E*_s_ to *E*_t_ at the channel height of 20 nm. (b) The relationship between *E*_s_ and the carbon number of n‐alkanes. (c) Schematic diagram of the *E*_s_ changing with the number of contact layers. (d) Comparison of the apparent viscosity obtained from MD and experimental methods. (e) Velocity distribution of confined fluids.

To further explore the intermolecular interactions at the fluid/channel wall interface, the average interaction energies (AIEs) between fluid molecules and channel walls (*E_s_*) were calculated for various fluids (water, C_6_H_14_, C_10_H_22_, C_12_H_26_, and C_16_H_34_) under different temperature and surface roughness states. Concurrently, the corresponding intermolecular interactions between the fluid (*E_i_*) were determined. These results are detailed in Section S11 (Tables S2 and S3). Figure 4a illustrates that the *E_s_* of different fluids accounted for a maximum of 31.17% of the total average interaction energy (*E_t_*), with the remaining portion being fluid intermolecular interactions. Additionally, the *E_s_* of fluids exhibited a gradual decrease as the distance from the wall increased, which is shown in Figure 4c. Furthermore, both *E_s_* and *E_t_* of alkane molecules simultaneously increased with carbon number, resulting in a less pronounced effect of carbon number on the *E_s_*/*E_t_* ratio for alkanes, as illustrated in Figure 4a. The exponential relationship between *E_s_* and carbon number was revealed in Figure 4b. The impact of temperature on intermolecular interactions was presented in Table S2. Findings indicated that both *E_i_* and *E_t_* decreased with increasing temperature, which corroborated the conclusion drawn from Figure 1a–d, where the apparent viscosity of fluids decreased with rising temperature. From a molecular perspective, fluid viscosity fundamentally originates from intermolecular interactions. Specifically, during thermal motion, strong interactions between adjacent flow layers in the interfacial region impede their relative movement. The molecules in the fast‐moving layer exert an attractive force on those closer to the wall (in the slow‐flowing layer), and conversely, the molecules in the slow‐moving layer hinder the faster ones. Due to their straight‐chain structure, alkane molecules exhibited a higher surface concentration of interaction energy with the wall compared with water (Figure S21). Furthermore, the probability of entanglement between long‐chain alkane molecules increased with carbon number, impeding the transport of alkane molecules. This effect is expected to be even more pronounced for isoalkanes (Figure S22).

Direct measurement of meniscus displacement becomes challenging for channels with heights below 70 nm, introducing some uncertainty into the data (See Section S12). For such cases, we developed a momentum exchange method as an alternative approach to determine apparent viscosity at the channel heights of 20 and 10 nm. As illustrated in Figure 4d, the apparent viscosity of water and C_16_H_34_ increased by 12.8% and 22.8% when the channel height was reduced from 20 to 10 nm. The observed trends across various fluids were found to be in close agreement with experimental measurements (Figure 1a–d). Further investigation into the apparent viscosity of fluids in smooth and rough nanochannels under varying temperatures was conducted. Results revealed that the apparent viscosity of water, C_6_H_14_, C_10_H_22_, C_12_H_26_, and C_16_H_34_ (channel height: 20 nm) decreased by 11.05%, 17.4%, 23.16%, 22.08%, and 23.91% when the surface transitioned from rough to smooth (Figure S23). This difference can be explained by the inherent structural properties of water vs. alkanes: water's simple tetrahedral molecular structure makes the *E_s_* less vulnerable to variations in channel surface roughness. In contrast, alkane molecules, particularly those with longer carbon chains, interact more significantly with the channel wall due to their larger contact area. Consequently, these structures are more affected by changes in the wall's topography or micro‐roughness features when transitioning between smooth and rough surfaces. As shown in Figure S24, the rough wall surface disrupted the parallel arrangement of alkane molecules near the channel boundary, resulting in changes to their interfacial molecular organization and increasing both the interfacial thickness as well as the viscosity. The apparent viscosity of the fluids decreased by 2.49%, 5.34%, 5.20%, 4.94%, and 5.10% when the temperature was increased from 30°C to 50°C (Figure S23). Furthermore, the reduction in *E_s_* of alkanes was more pronounced with increasing carbon number (Figure S25). For instance, the *E_s_* reduction of C_16_H_34_ on a silica surface reached 51.03% when transitioning to a smooth surface configuration. The velocity distribution of fluids in the nanochannel exhibited significant disparities between the interfacial layer and the bulk region (Figure 4e). These variations in velocity profiles might be primarily attributed to distinct hydrodynamic slip behaviors, which resulted from differences in apparent viscosity among various fluids. With regard to C_6_H_14_, the peak flow velocity was significantly higher due to its lower viscosity, minimizing surface friction. In contrast, C_16_H_34_ exhibited a much slower peak velocity of only 38.1% as compared with C_6_H_14_, which was mainly attributed to greater fluid resistance.

This study systematically investigated the apparent viscosity behavior of fluid in nanoconfined space ranging from 3 to 1000 nm. By combining experimental measurements and MD simulations, we proposed a mathematical model to characterize the interfacial layer properties of confined fluid. This advanced model, by incorporating specific material properties and temperature conditions, and fluid characteristics describes the interfacial layer thickness and viscosity of fluid in confined spaces below 20 nm. The molecular interaction coefficient (*f_i_*) in the model exhibited a strong linear correlation with the carbon number of n‐alkanes, while it was less affected by channel width and highly sensitive to temperature.

We have uncovered key aspects of how fluid viscosity was profoundly influenced by nanoscale confinement. The interfacial layer exhibited a viscosity enhancement factor of 6.89 times compared to the bulk state when the channel height was 70 nm, establishing it as the primary determinant of the anomalous transportation of nanoconfined fluid. Further analysis revealed that significant intermolecular forces in the interfacial layer accounted for 31.17% of the total interaction energy, illuminating the fundamental molecular‐scale origin of abnormal viscosity behavior. Both fluid molecular structure and wall roughness significantly influenced the molecular alignment patterns and interfacial layer thickness. Surface smoothing reduced average interaction energy by 24.6% and apparent viscosity by 18.3% in comparative trials.

## 4 Materials and Methods

### 4.1 Device Fabrication

**FIGURE 5:**
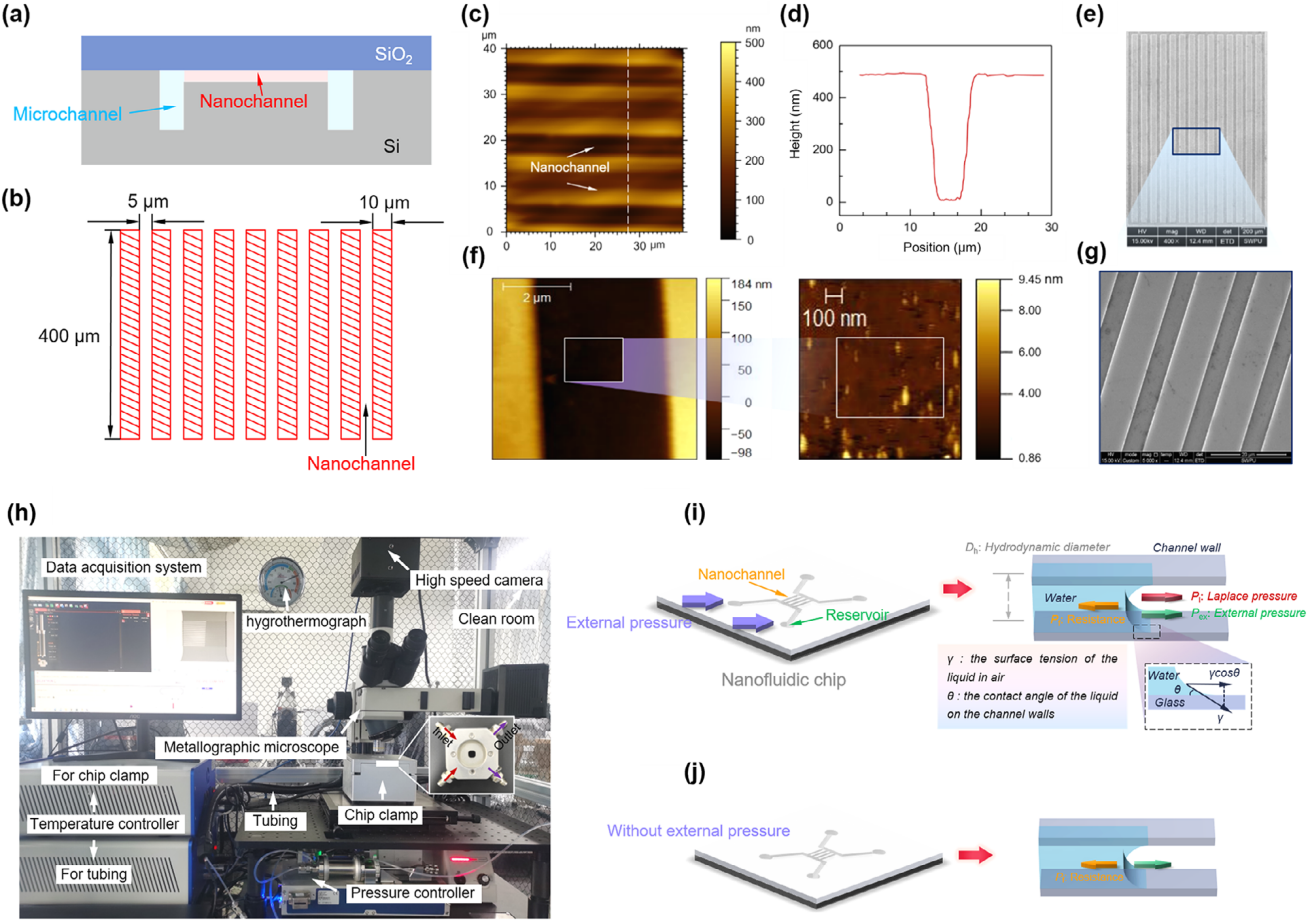
The parameters and characterization of the nanofluidic device for apparent viscosity study. (a) Schematic of the nanofluidic chip: the substrate material (silicon) and cover material (glass) were bonded by anodic bonding technology. (b) The size parameters of nanochannels. (c) The AFM morphology of nanochannels. (d) Typical AFM results of a nanochannel with a width of 5 µm and a height of 500 nm. The SEM morphology of the nanochannels at 400× (e) and 5000× (g). (f) The surface roughness test results of the nanochannel with a height of 500 nm. (h) Schematic illustration of a nanofluidic setup. Fluids filling speed method for (i) with external pressure and (j) without external pressure.

The nanofluidic chip was composed of micro‐and nanochannels. The substrate and upper surface material were silicon and glass, as presented in Figure 5a. There were 12 parallel nanochannels placed perpendicularly along the microchannel, and each channel was 5.0 µm in width and 400 µm in length (Figure 5b). This identical layout was applied for chips with the height of 20, 50, 70, 130, 200, 300, 500, 800, and 1000 nm. The inlet and outlet holes were symmetrically distributed at two ends of the microchannels. The fabrication procedure of the nanofluidic chip was described in Section S1. Atomic force microscope (AFM) and scanning electron microscope (SEM) were used to verify the machining accuracy of nanofluidic chips (Figure 5c–g). As seen, the average machining error of a nanochannel with a depth of 500 nm was approximately 1.3 nm (Figure 5d), and the average surface roughness of the nanochannel was 0.22 nm (Table S1). No defects were observed on the nanochannel surface according to the SEM images (Figure 5g).

### 4.2 Fluid Transportation

The fluid flow in the nanofluidic chip included driving and imbibition in this study. The nanofluidic chip was installed on a pressure clamp (0–500 bar) and connected with a micro‐pump (CETONI‐Nemesys middle pressure module, 0–22 ± 0.01 bar, 0–100 ± 0.01°C). The tubing and chip clamp were placed in a heated band and a heated box, respectively. The temperature was regulated by a temperature controller (TCG100, 0–100 ± 0.01°C). A high‐speed camera (Photron‐Fastcam Mini UX50, max FPS 12000) connected to a metallographic microscope (BA310MET‐H, Motic) with a 10× objective was used to visualize the fluid transportation and record the fluid meniscus fronts in the testing area of the nanochannels. The whole testing system was placed in a clean room (Figure 5h). The micro‐pump, tubing, and valve were completely cleaned before connecting with the nanofluidic chip.

For the driving process (Figure 5i), the tubing and chip clamp were first heated to 30°C for 60 min to achieve a stable thermal state before introducing deionized water (electrical resistivity: 16.23 MΩ·cm) into the nanofluidic chip. The controlled pressure maintained a constant flow of fluid through the nanochannels, which was recorded by the high‐speed camera. The video of fluid transportation in the nanochannels was disassembled into images using MATLAB software. The apparent viscosity of water was determined by establishing the correlation between the displacement of the fluid meniscus (Δ*x*), time difference (Δ*t*), and external pressure (*P_ex_*), as shown in Section S3. Slight variations in nanochannel roughness among different chips existed on the same wafer (See Section S2, Table S1) due to processing limitations. The reusability of the nanofluidic chips was investigated, as depicted in Section S2. Subsequently, temperature (40°C, 50°C) and fluid type (n‐Alkanes‐98% pure and ethanol‐95% pure obtained from Sigma–Aldrich) were changed to repeat the above steps. All the fluids were degassed before the experiments. For the imbibition process (Figure 5j), a liquid drop was placed on the injection hole of the nanofluidic chip. The imbibition process was then spontaneously launched by infiltrating the drop into the nanochannels, as captured by the high‐speed camera. The entire system, including the working fluid, was equilibrated for 60 min to ensure a uniform thermal state before the experiments. To assess the possible presence of interfacial thermal resistance, the water was continuously injected at 30°C, 50°C, and 80°C, while the temperatures at the chip inlet and outlet were simultaneously monitored. All data presented in this paper are expressed as the average values of three replicate experiments. The data uncertainty is estimated to be 5%, based on the observation accuracy of the high‐speed camera.

### 4.3 Molecular Dynamics Simulation

To investigate the interactions between fluid molecules and channel wall in nanoconfined space, nanochannels formed by silicon and silica molecules were set (channel height: 3, 5, 10, 20, 40, and 70 nm) using the classic molecular simulation method (See Section S8). The upper and lower surfaces of the channel were configured to have a defective state with roughness values of 0.2832 and 0.2640 nm, respectively (Figure S17), closely resembling the roughness of the nanochannel surface materials in this study (silica: 0.2406 nm, silicon: 0.2202 nm). All the molecular dynamics simulations were performed utilizing the LAMMPS software package [50]. To explore the effect of wall roughness on the intermolecular interactions, a smooth nanochannel was used as the control group (Figure S18). The molecular numbers of five fluids in the simulation channel were set to 52 000, 7400, 5800, 4200, and 3000, respectively, and randomly distributed in the simulation channel based on the bulk density. After constructing the initial bound state of fluid molecules, the channel surface was set in a rigid state to maintain the substrate atoms in a fixed state during the simulation process. The fundamental theories for calculating intermolecular interaction force, viscosity, and density of nanoconfined fluids were described in Sections S9 and S10.

## Author Contributions

X.Z., B.W., and R.‐N.W. contributed to the investigation. X.Z., B.W., and R.‐N.W. contributed to methodology. B.W. and J.‐Y.W. contributed to supervision. X.Z., B.W., and J.‐Y.W. contributed to writing, reviewing, and editing.

## Conflicts of Interest

The authors declare no conflicts of interest.

## Supporting information




**Supporting File**: advs75496‐sup‐0001‐SuppMat.docx.

## Data Availability

The data that support the findings of this study are available on request from the corresponding author. The data are not publicly available due to privacy or ethical restrictions.
