# Supplementary material for: Advanced Multiscale Modeling for Revealing Anomalous Fluid Transport Induced by Confinement Interfacial Layer Reconstruction in Sub‐10 nm Space
Source: Adv Sci (Weinh). 2026 Apr 29;13(41):e75496. doi: 10.1002/advs.75496 (PMC13335607; doi:10.1002/advs.75496)
Supplement: Supplementary file 1 — Supporting File: advs75496‐sup‐0001‐SuppMat.docx. [file ADVS-13-e75496-s001.docx]

**Supporting information**

**Advanced Multiscale Modeling for Revealing Anomalous Fluid Transport Induced by Confinement Interfacial Layer Reconstruction in sub-10 nm Space**

Xiang Zhang^1, 2, 3^, Bing Wei^1,^[[1]](#footnote-1)^⁎^, Jingyi Wang^4^, Runnan Wu^1^

*1) State Key Laboratory of Oil and Gas Reservoir Geology and Exploitation, Southwest Petroleum University, Chengdu, 610500, China*

*2) Department of Energy and Power Engineering, Tsinghua University, 100084, China*

*3) Department of Building Environment and Energy Engineering, The* *Hong Kong Polytechnic University, Hong Kong, 999077, China*

*4)* *College of Chemistry and Chemical Engineering, Southwest Petroleum University, Chengdu, 610500, China*

# Sections

S1. Nanofluidic device fabrication procedure

S2. The reusability testing of nanofluidic chips

S3. The apparent viscosity measurements of nanoconfined fluids

S4. The interfacial layer thickness of nanoconfined fluids

S5. The calculation theories of the viscosity in the interfacial layer

S6. The density distribution of nanoconfined fluids in a nanochannel

S7. The interfacial layer thickness model of different fluids

S8. Basic parameters in a molecular simulation study

S9. The calculation principle of intermolecular interaction forces

S10. The calculation principle of fluid viscosity and density

S11. The average interaction energy (AIE) of nanoconfined fluids

S12. Nanobubbles in the fluid imbibition process

# S1. Nanofluidic device fabrication procedure


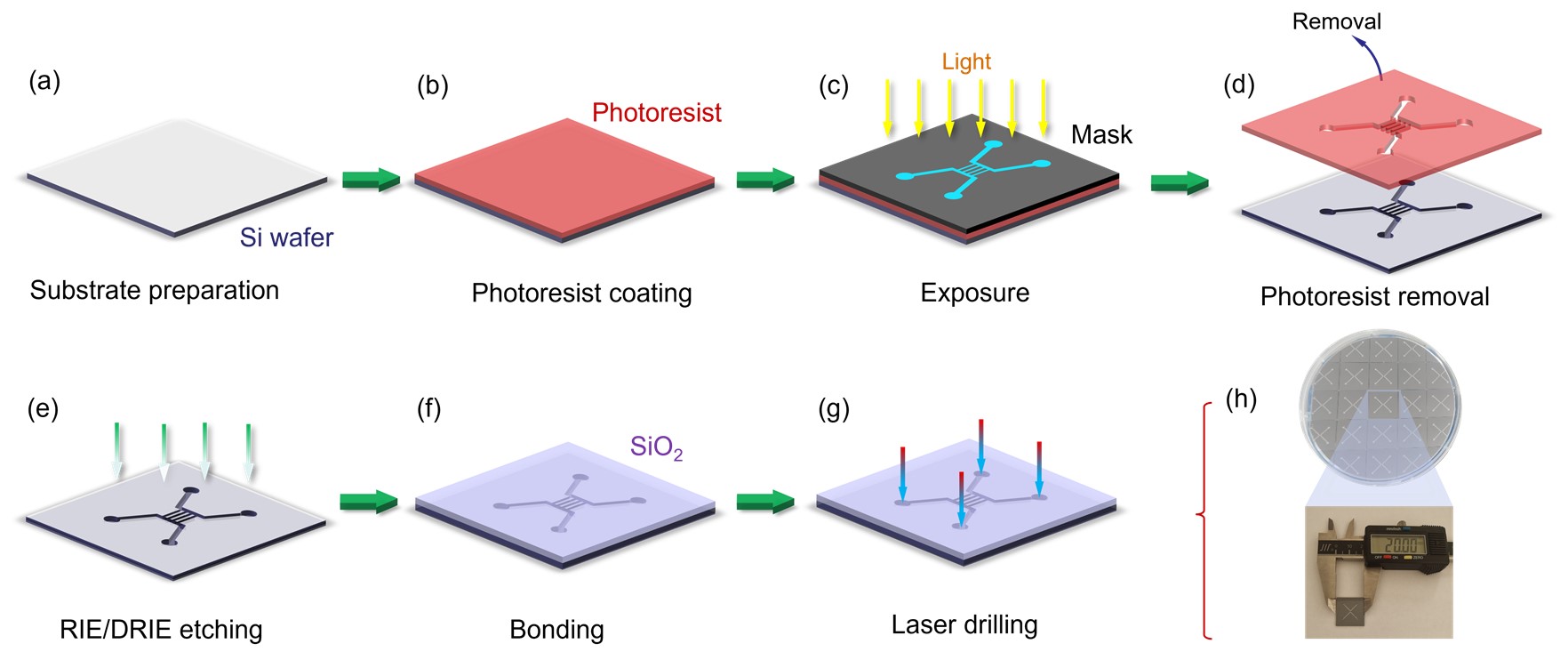


**Figure S1. Fabrication procedure of the nanofluidic device. (a)** Clean and dry the substrate material (silicon wafer). **(b)** Coat the photoresist on the silicon wafer. **(c)** Coat the mask on the surface of the photoresist, followed by exposure and development. **(d)** Remove the photoresist and mask. **(e)** The RIE/DRIE etching method was applied to form microchannels and nanochannels. **(f)** The substrate material (silicon) and cover material (glass) were bonded by anodic bonding technology. **(g)** The injection pores on the glass were produced by laser drilling technology. **(h)** Image of the nanofluidic chips.

# S2. The reusability testing of nanofluidic chips

The cleaning steps of nanofluidic chips are described as follows: (1) Soak the nanofluidic chip in ethanol and clean it with an ultrasonic instrument (SN-QX-100) for 30 minutes. (2) Dry the nanofluidic chip with high-purity nitrogen and soak it in acetone, then clean it with an ultrasonic instrument for 30 minutes. (3) Dry the nanofluidic chip with high-purity nitrogen and test the fluid apparent viscosity (Figure S2a). The results indicated that the maximum fluctuation of water apparent viscosity was only 0.0014 mPa·s after 20 times of experiments and cleaning procedures (Figure S2b), namely, repeated cleaning would not affect the surface properties of the nanofluidic chip.


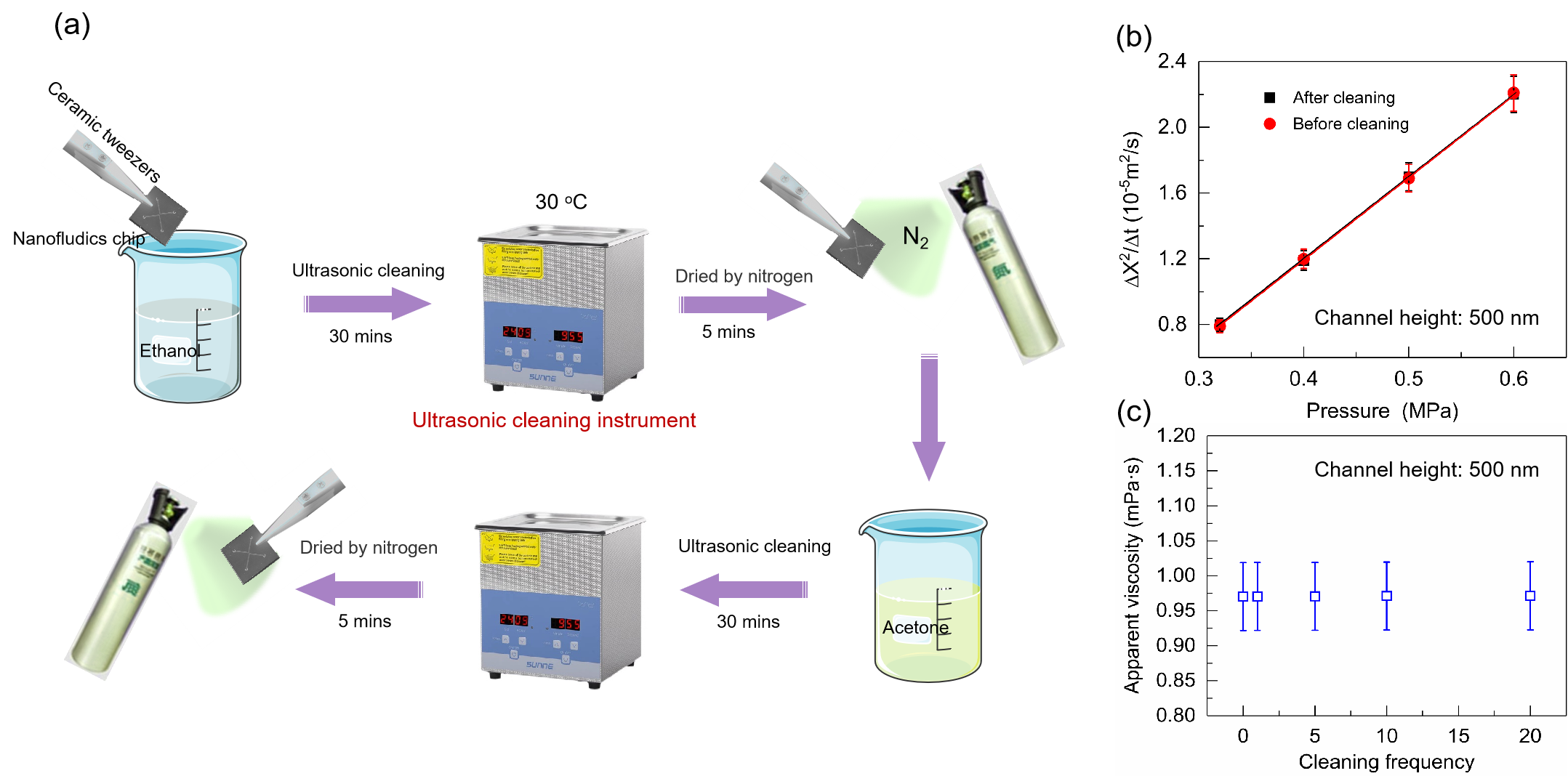


**Figure S2. Cleaning of the nanofluidic chip. (a)** The cleaning procedures of the nanofluidic chip. **(b)** The relationship between Δ*x*^2^/Δ*t* and external pressure. **(c)** The relationship between nanofluidic chip cleaning frequency and water apparent viscosity in the nanochannel with a height of 500 nm at 30 ^o^C.

**Table S1** The average surface roughness of different nanofluidic chips on a wafer

| Chip number | 1 | 2 | 3 | 4 | 5 | 6 | 7 | 8 |
| --- | --- | --- | --- | --- | --- | --- | --- | --- |
| Avg. roughness, nm | 0.2185 | 0.2202 | 0.2193 | 0.2214 | 0.2208 | 0.2211 | 0.2224 | 0.2216 |

# S3. The apparent viscosity measurements of nanoconfined fluids

The fluid filling speed in a nanochannel can be characterized by the classical Washburn model^[1]^, which relies on the equilibrium between the capillary force sucking the fluid into the channel and the resistance opposing the liquid motion. To avoid using unknown *γ* and *θ* for viscosity calculation, a pressurized capillary flow method was used^[2]^. When the external pressure *P*_ex_ was applied in the nanoconfined fluid, the pressure balance of the capillary filling in the steady state could be described as:

$P_{l}+\Delta P_{v}=P_{c}+P_{ex}$ (S1)

where *P*_l_ and Δ*P*_v_ are the inertial and viscous forces along the channel for fluid, respectively. *P*_c_ denotes the capillary force of fluid. The fluid inertia force could be ignored in the nanochannels. Thus, the Eq. S1 could be rewritten as:

$\Delta P_{v}=P_{c}+P_{ex}$ (S2)

The fluid viscous force can be described as:

$P_{v}=Gx=\frac{\Delta P}{\Delta x}x$ (S3)

The flow pattern of fluid in nanochannels was assumed to be laminar flow, and the viscous force predominantly contributed to the flow resistance. The flow rate near the channel surface was assumed to be zero, and the average flow rate of fluid in the rectangular channel could be expressed as^[3]^:

$v_{m}=\frac{dx}{dt}=G\left(\frac{8h^{2}}{\pi^{4}\mu}\right)\sum_{n=0}^{\infty} \frac{1}{\left(2n+1\right)^{4}}\left[1-\frac{1}{r\left(n\right)}\tanh r\left(n\right)\right]$ (S4)

And *r*(*n*) can be described as $r(n)=(2n+1)\frac{\pi w}{2h}$, depending on the channel aspect ratio of $\varepsilon=\frac{h}{w}$ and *n*. The calculation error was found to be less than 4% under the condition of $0<\varepsilon<1.2$ if using only n = 0, neglecting terms for n = 1 and higher. Thus, we used only the term n =0 for simplicity, and Equation S4 can be written as:

$v_{m}=G\left(\frac{8h^{2}}{\pi^{4}\mu}\right)\left[1-\frac{1}{r\left(0\right)}\tanh r\left(0\right)\right]$ (S5)

Integrating equation S3 and S5 yields:

$\Delta P_{V}=\frac{\pi^{4}\mu}{8h^{2}\left[1-\frac{2h}{\pi w}\tanh\left(\frac{\pi w}{2h}\right)\right]}x\frac{dx}{dt}$ (S6)

The *P*_c_ can be written as:

$P_{c}=2\gamma\cos\theta\left(\frac{1}{w}+\frac{1}{h}\right)=\frac{4\gamma\cos\theta}{D_{h}}$ (S7)

And

$D_{h}=\frac{2wh}{w+h}$ (S8)

Integrating equation S2, S6-S8 yields:

$\frac{4\gamma cos\theta}{D_{h}}+P_{ex}=\frac{\pi^{4}\mu}{8h^{2}\left[1-\frac{2h}{\pi w}\tanh\left(\frac{\pi w}{2h}\right)\right]}x\frac{dx}{dt}$ (S9)

The relationship between the displacement of the fluid meniscus (Δ*x*) and the time difference (Δ*t*) can be expressed as follows:

$\frac{\Delta x^{2}}{\Delta t}=\frac{16h^{2}\left[1-\frac{2h}{\pi w}\tanh\left(\frac{\pi w}{2h}\right)\right]}{\pi^{4}\mu}\left(\frac{4\gamma\cos\theta}{D_{h}}+P_{ex}\right)$ (S10)

where *G* is the pressure gradient, Δ*p* represents the pressure difference between two points separated by distance Δ*x, v*_m_ denotes the average velocity, *h* and *w* stand for the height and width of the nanochannel, *μ* is the fluid viscosity, *γ* is the interfacial tension between two phases, *θ* is the contact angle, *D*_h_ stands for the hydraulic radius of the nanochannel. The measurements of the fluid apparent viscosity procedure are as follows: (1) Record the meniscus displacement (Δ*x*) and time (Δ*t*) of water in nanochannels under different pressures (0.32 MPa, 0.40 MPa, 0.50 MPa, 0.60 MPa) (Figure S3a); (2) Calculate the slope (*k*) of Δ*x*^2^ and Δ*t* under different pressures, and establish the relationship between *k* and external pressure (*P*_ex_) (Figure S3b). The apparent viscosity of water in the channel height ranged from 70 to 1000 nm, and channel width of 5 μm and 15 μm was obtained, as shown in Figure S3c.


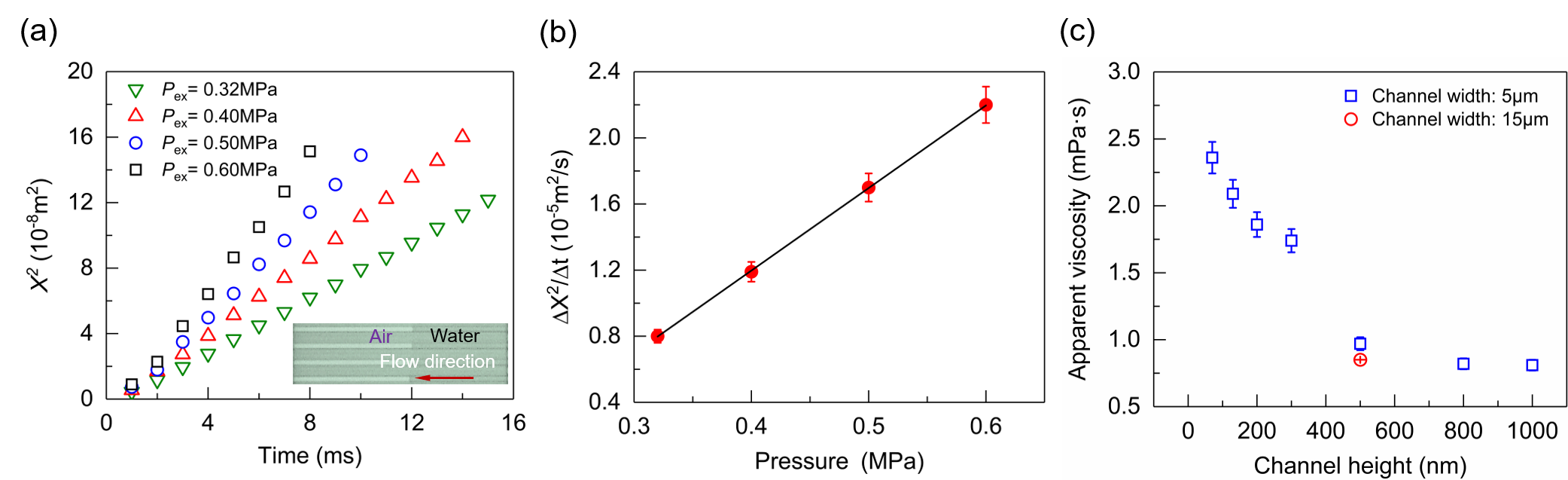


**Figure S3. Calculation produces for the fluid apparent viscosity. (a)** The relationship between Δ*x*^2^ and Δ*t* under different pressures. **(b)** The relationship between Δ*x*^2^/Δ*t* and external pressure. **(c)** The apparent viscosity of water in a nanochannel with a height ranging from 70 nm to 1000 nm and a width of 5 μm and 15 μm at 30 ^o^C.

# S4. The interfacial layer thickness of nanoconfined fluids

The mass flow of nanoconfined fluid during the imbibition process can be expressed as follows:

$Q=\frac{\Delta p}{\zeta}=wh\frac{dx}{dt}$ (S11)

The laminar flow model was used to describe the fluid transport process since the Reynolds number of the fluid during the imbibition process was relatively small^[4]^. According to the Hagen-Poiseuille equation, the flow resistance of a plane flow can be expressed as follows:

$\xi=\frac{12\mu x}{wh^{3}}$ (S12)

The flow resistance of fluid in the interfacial layer was much greater than that in the bulk region. Thus, the flow resistance of nanoconfined fluid can be rewritten as follows:

$\xi_{a}=\frac{12\mu x}{w\left(h-2h_{a}\right)^{3}}$ (S13)

The width of nanochannels in nanofluidic chips was much greater than the depth in this study. Therefore, the capillary pressure of fluid in a nanochannel can be expressed as follows:

$\text{Δ}p=\frac{2\gamma cos\theta}{h}$ (S14)

The classical L-W equation ($x^{2}=\left( \frac{\gamma hcos\theta}{3\mu} \right)t$) can be rewritten as $x^{2}=At$($A=\frac{\gamma hcos\theta}{3\mu}$). The L-W equation obtained from the experimental data can be expressed as:

$x^{2}=A_{exp}t$ (S15)

Combining the formulas S11-S13 and the L-W equation, the deviations of imbibition flow in nanochannels can be obtained:

$\frac{A_{exp}}{A_{theo}}=\left(1-\frac{2h_{a}}{h}\right)^{3}$ (S16)

The $\frac{A_{exp}}{A_{theo}}$ can be obtained by comparing the $\Delta x^{2}\propto\Delta t$ curve between the experimental data and predicted data by the L-W equation (Figure 3c and 3d). Therefore, the interfacial layer thickness can be obtained through formula S16.

In Equations S11-16, *Q* is the mass flow of fluid, Δ*p* stands for the capillary force of fluid, *ζ* represents the flow resistance of fluid, *w* and *h* are the width and width of the nanochannel respectively, *x* denotes the the meniscus displacement of fluid, *t* is the elapsed time, *θ* is the contact angle, *μ* represents fluid viscosity, *γ* denotes the air-liquid surface tension, *ζ*_a_ is the flow resistance of fluid considering the interfacial layer, and *h*_a_ stands for the interfacial layer thickness.


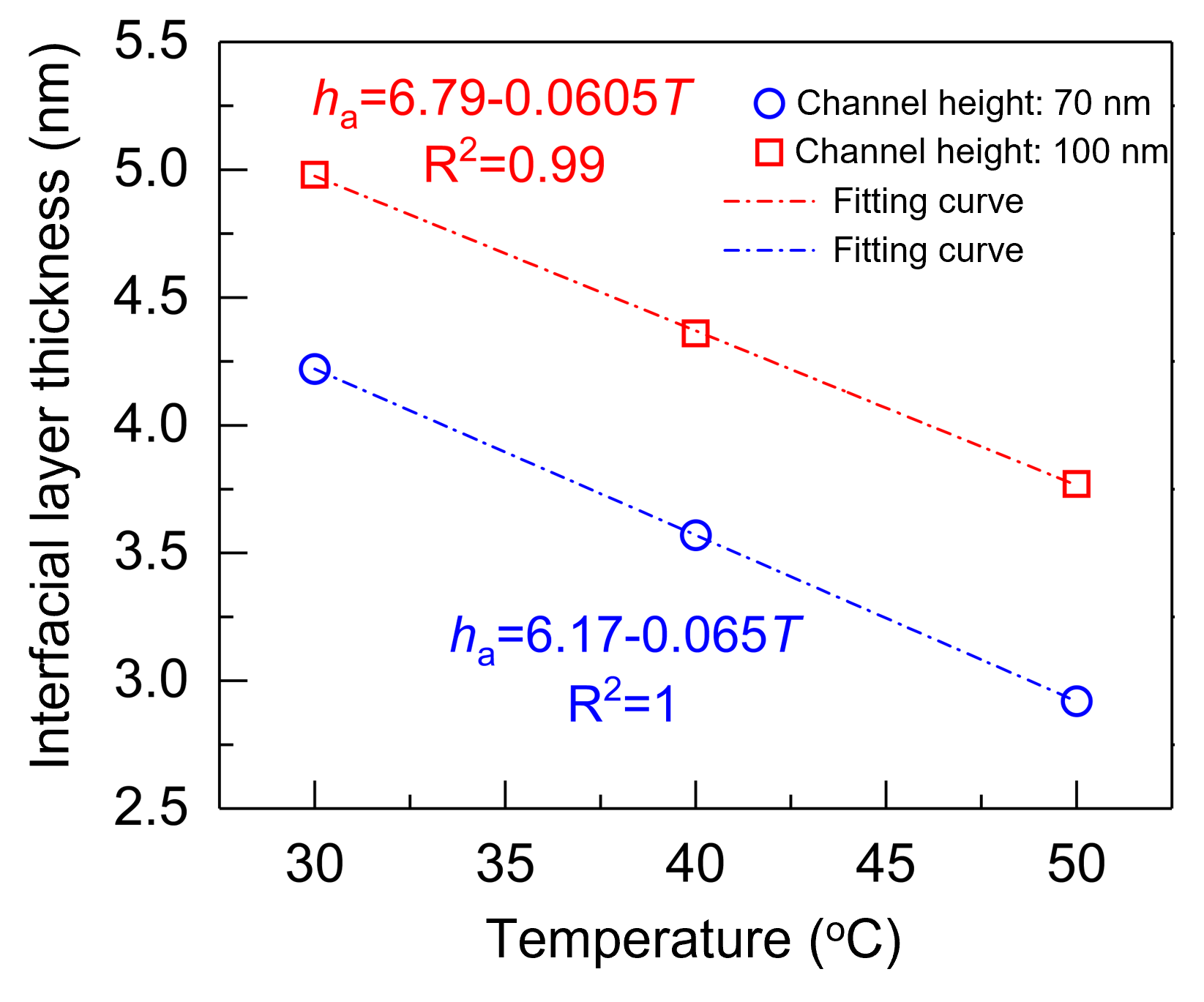


**Figure S4.** The correlation between the water interfacial layer thickness and temperature in the nanochannel with a height of 70 nm and 100 nm.

# S5. The calculation theories of the viscosity of nanoconfined fluid in the interfacial layer

Based on Figure 2a, assume the *μ*_f_ to be the viscosity in the interfacial layer with a thickness of *h*_a_ and the *μ*_0_ to be the bulk viscosity. The apparent fluid viscosity in the interfacial layer *μ*_f_ can be calculated by the following equation^[5]^.

$A_{exp}=\sqrt{\left(\frac{8\gamma\kappa}{h}\right)\left[\left(\frac{1}{\mu_{0}}-\frac{1}{\mu_{f}}\right)\left(\frac{h_{a}^{3}}{3}\right)+\frac{1}{\mu_{f}}\frac{h^{3}}{24}\right]}$ (S17)

And *κ* can be obtained as^[6]^:

$\kappa=\frac{1}{2}\left(\frac{1}{h}+\frac{1}{w}\right)cos\theta$ (S18)

where *κ* is the mean curvature of the interface.

Thus, the *A*_exp_ corresponding to the channel height could be calculated by the fluid imbibition experiment, thereby obtaining the apparent fluid viscosity in the interfacial layer *μ*_f_.


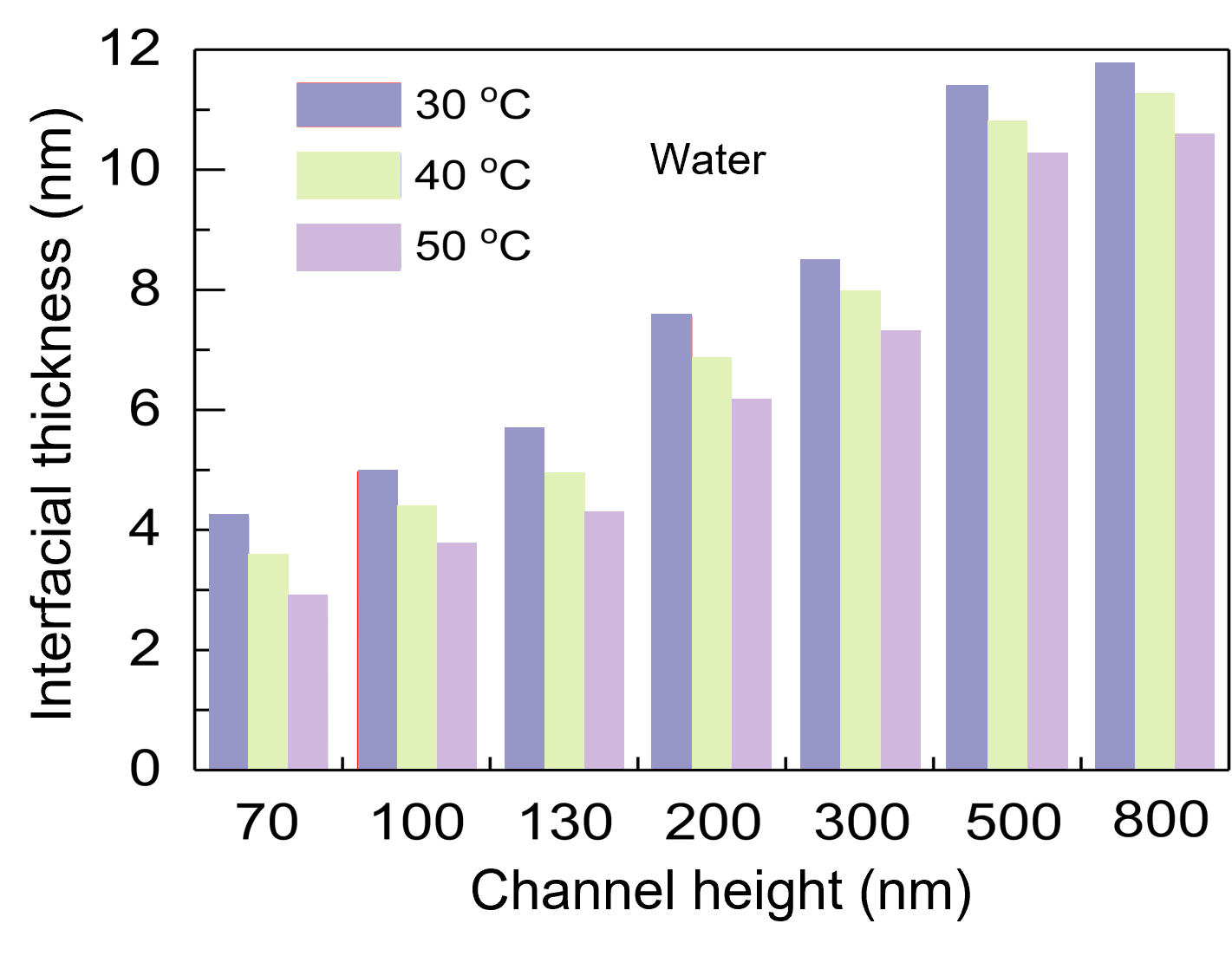


**Figure S5.** The interfacial thickness of water with channel height in the imbibition process under different temperatures.

**
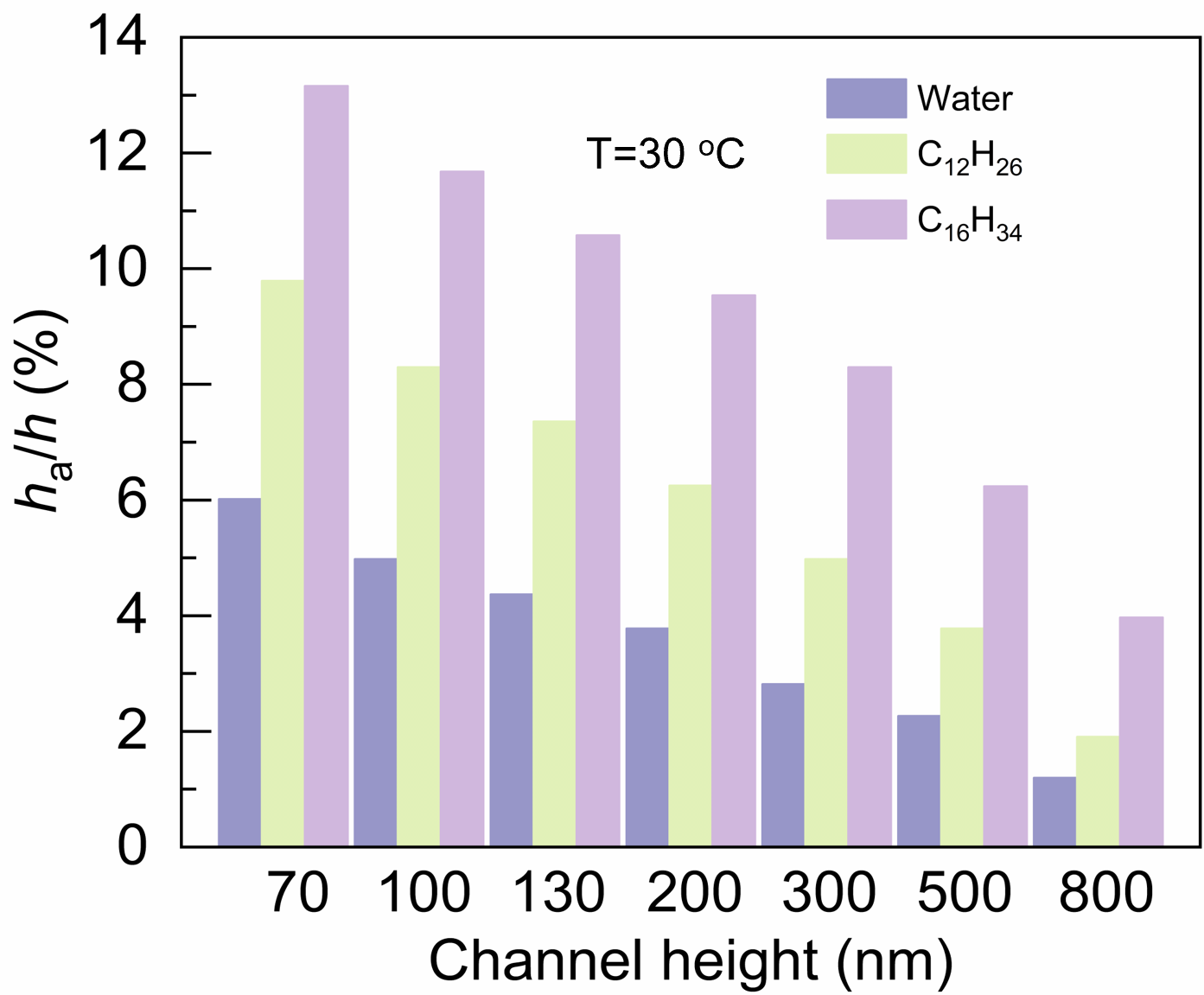
**

**Figure S6.** The ratio of interfacial thickness of nanoconfined fluids to channel height as a function of channel height.


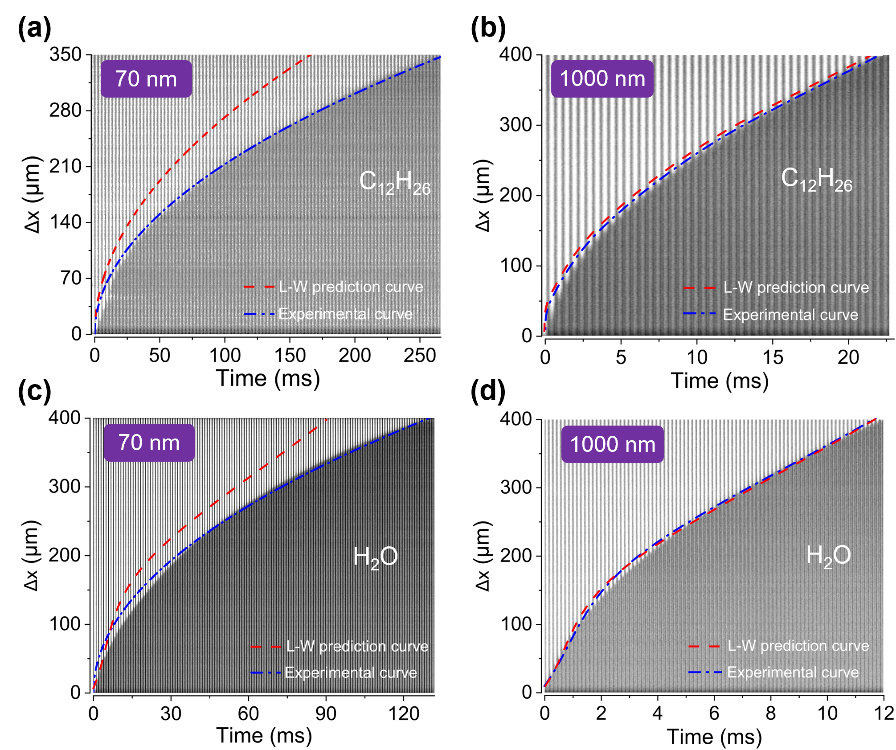


**Figure S7.** Comparison of L-W model prediction curves and experimental data for H_2_O and C_12_H_26_ during imbibition process at different scales.

# S6. The density distribution of nanoconfined fluids in a nanochannel

The density distribution fluctuations within the interfacial regions exhibited pronounced variations, particularly in the silica wall-interaction area across different fluids compared to those in the silicon surface-interaction region. These findings underscore that the average interaction energy between fluid molecules and the silica surface was significantly higher than that with the silicon surface. The interaction boundary between alkane molecules and the channel wall was notably larger than that of water molecules, while the interaction boundary between fluids and the silica surface exhibited a greater value compared to the silicon surface (Figure S8). The maximum density of fluids (water, C_12_H_26_, and C_16_H_34_) in nanochannels relative to their bulk value showed ratios of 1.34, 2.04, and 2.12, respectively, aligning with the observed variation in fluid apparent viscosity ratios within the nanochannel compared to the bulk. Furthermore, the width of the fluid density fluctuation region decreased (Figure S9), and this reduction was closely associated with the specific characteristics of fluid type when the channel surface state transitioned from rough to smooth (Figure S10).


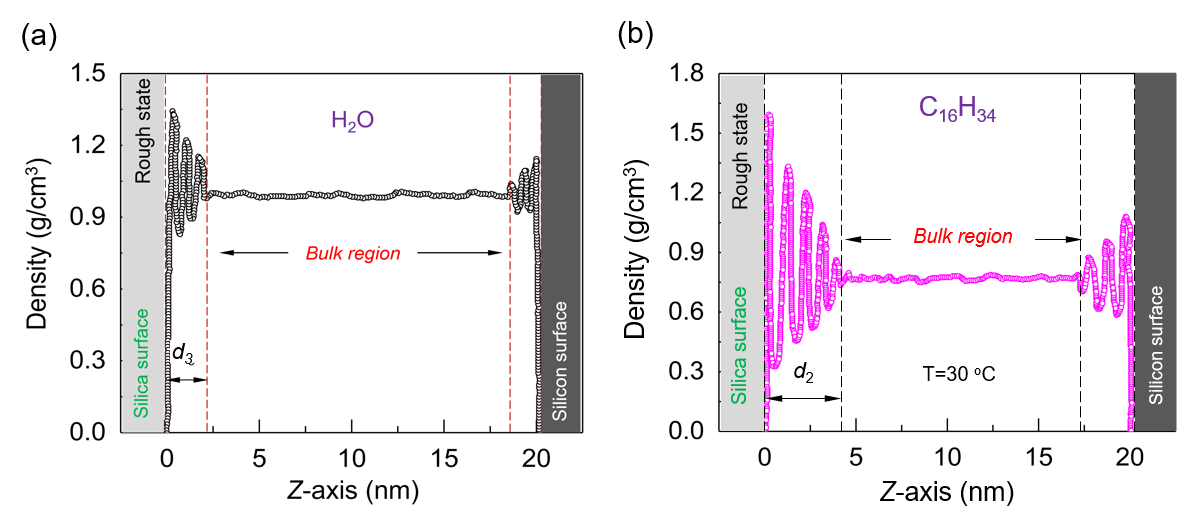


**Figure S8.** Density distribution of C_16_H_34_ **(a)** and water **(b)** in a nanochannel (width: 20 nm) with a rough surface.


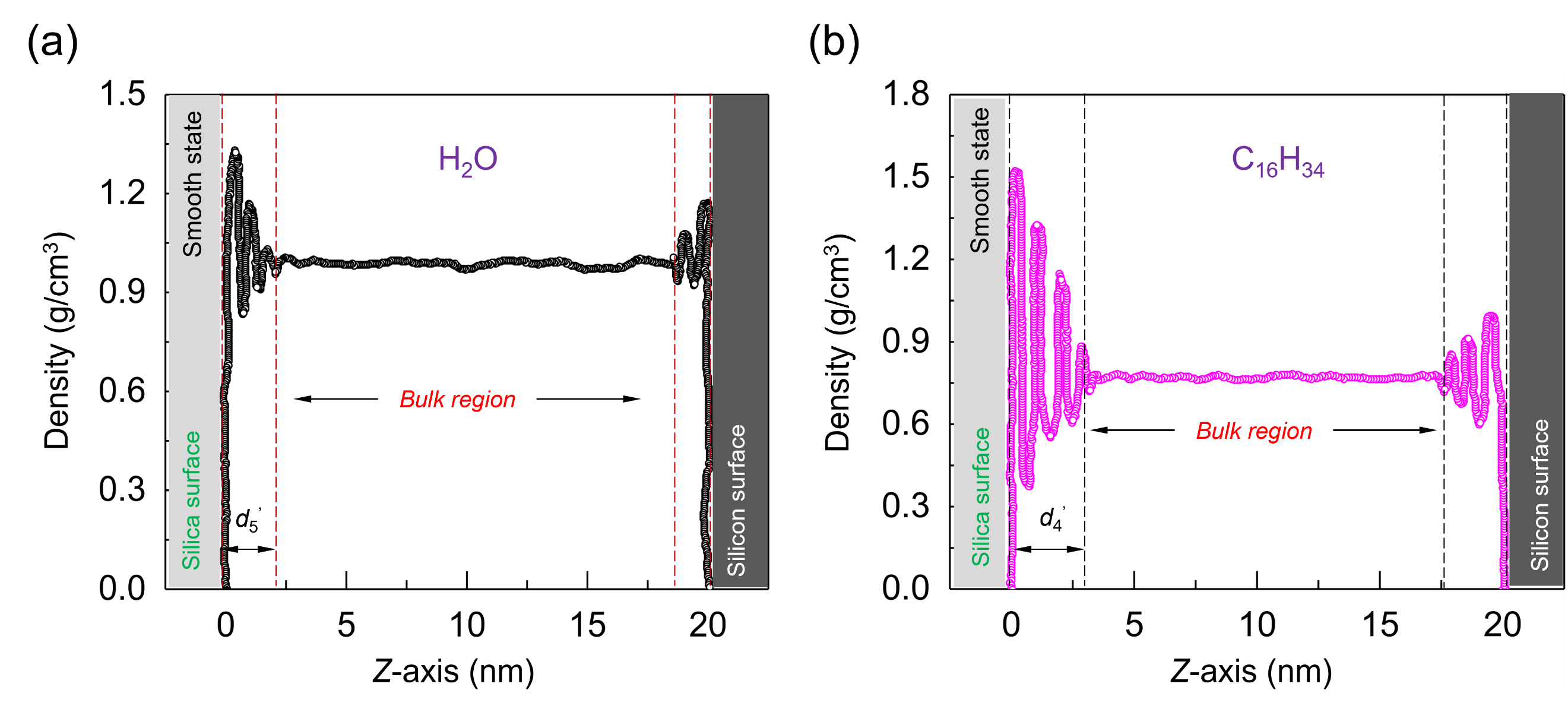


**Figure S9.** Density distribution of water **(a)** and **(b)** C_16_H_34_ in a nanochannel (width: 20 nm) with a smooth surface.


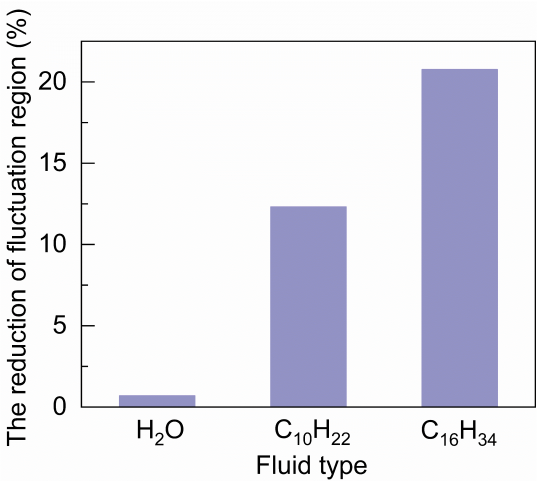


**Figure S10.** The reduction of the fluctuation region when the channel surface state changes from rough state to smooth state.

# S7 The interfacial layer thickness model of different fluids


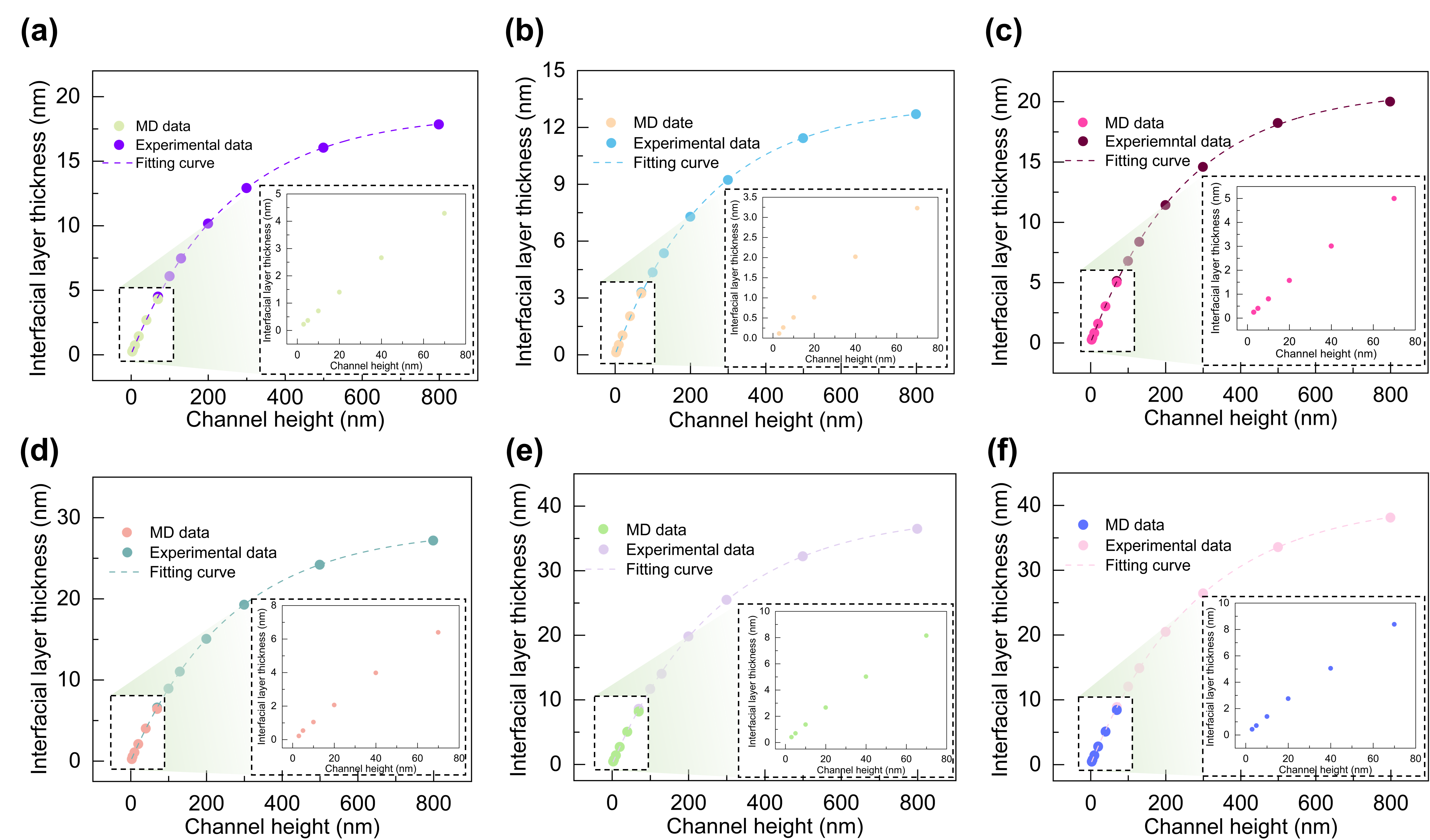


**Figure** **S11.** The interfacial layer thickness model of **(a)** ethanol, **(b)** C_8_H_18_, **(c)** C_12_H_26_, **(d)** C_14_H_30_, **(e)** C_17_H_36,_ and **(f)** C_18_H_38_ versus channel height.

The interfacial layer thickness model of different fluids in a nanoconfined space is expressed as follows:

$H_{i-ethanol}=18.65424\left(1-e^{-0.00393h}\right)$ (R^2^=99%) (S19)

$H_{i-C8\text{H}18}=13.24767\left(1-e^{-0.00398\text{h}}\right)$ (R^2^=99%) (S20)

$H_{i-C12\text{H}26}=20.02212\left(1-e^{-0.00381\text{h}}\right)$ (R^2^=99%) (S21)

$H_{i-C14\text{H}30}=28.62833\left(1-e^{-0.00373\text{h}}\right)$ (R^2^=99%) (S22)

$H_{i-C16H34}=36.20141\left(1-e^{-0.00362h}\right)$ (R^2^=99%) (S23)

$H_{i-C17\text{H}36}=38.68741\left(1-e^{-0.00358\text{h}}\right)$ (R^2^=99%) (S24)

$H_{i-C18\text{H}38}=40.56420\left(1-e^{-0.00353\text{h}}\right)$ (R^2^=99%) (S25)

Due to the limited accuracy of experimental observations, the errors associated with experimentally measured fluid transport parameters become relatively larger when the spatial scale falls below 70 nm. As the spatial scale decreases further, experimental characterization becomes exceptionally challenging. MD simulations were conducted to establish the relationship between meniscus displacement ($\Delta x$) and time ($t$) during the spontaneous imbibition of fluids in spaces smaller than 70 nm. These results were then incorporated into the following equation to determine the thickness of the interfacial fluid layer under sub-70 nm confinement. However, we found that the interfacial layer thickness predicted by this model from the simulation data exhibited no clear correlation with penetration depth. Its variation with spatial scale also appeared unreasonable, showing irregular fluctuations (Figure S12). These results suggest that the model is unable to accurately predict the thickness of the fluid interfacial layer under strong confinement, although the underlying cause remains unclear.

$$\frac{A_{exp}}{A_{theo}}=\left(1-\frac{2h_{a}}{h}\right)^{3}$$

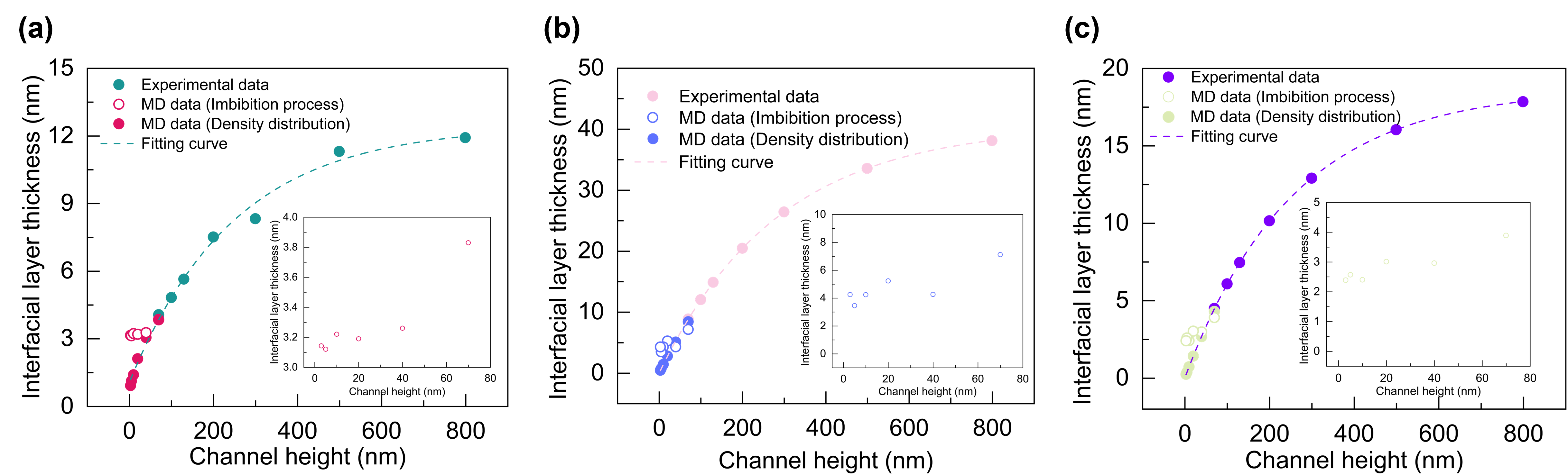


**Figure S12.** The interfacial layer thickness of confined fluids **(a)** water, **(b)** C_18_H_38_, and **(c)** ethanol obtained from self-imbibition simulation and density oscillation simulation under strong confinement conditions.


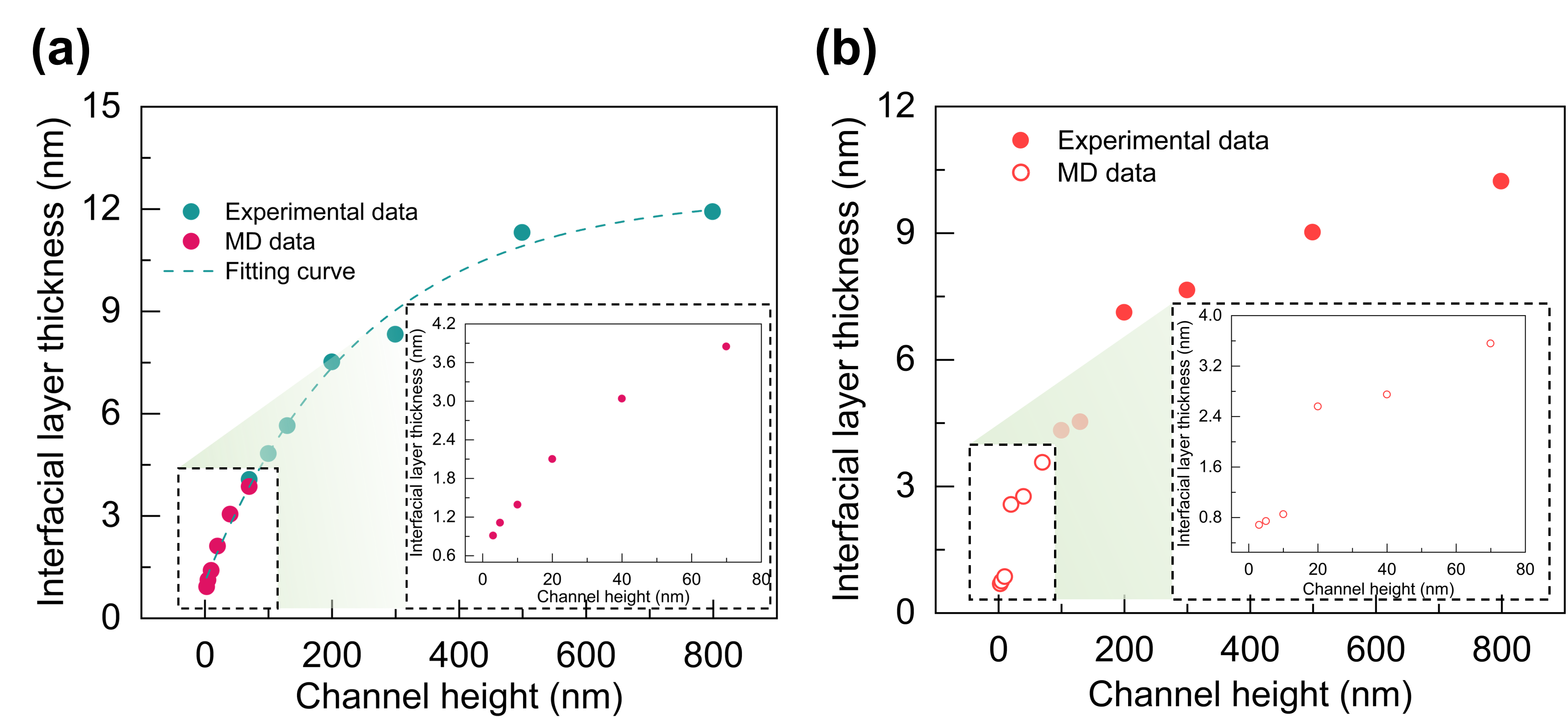


**Figure S13.** The relationship between the thickness of the interfacial layer and the channel height under different wetting conditions: **(a)** hydrophilic state and **(b)** hydrophobic state.


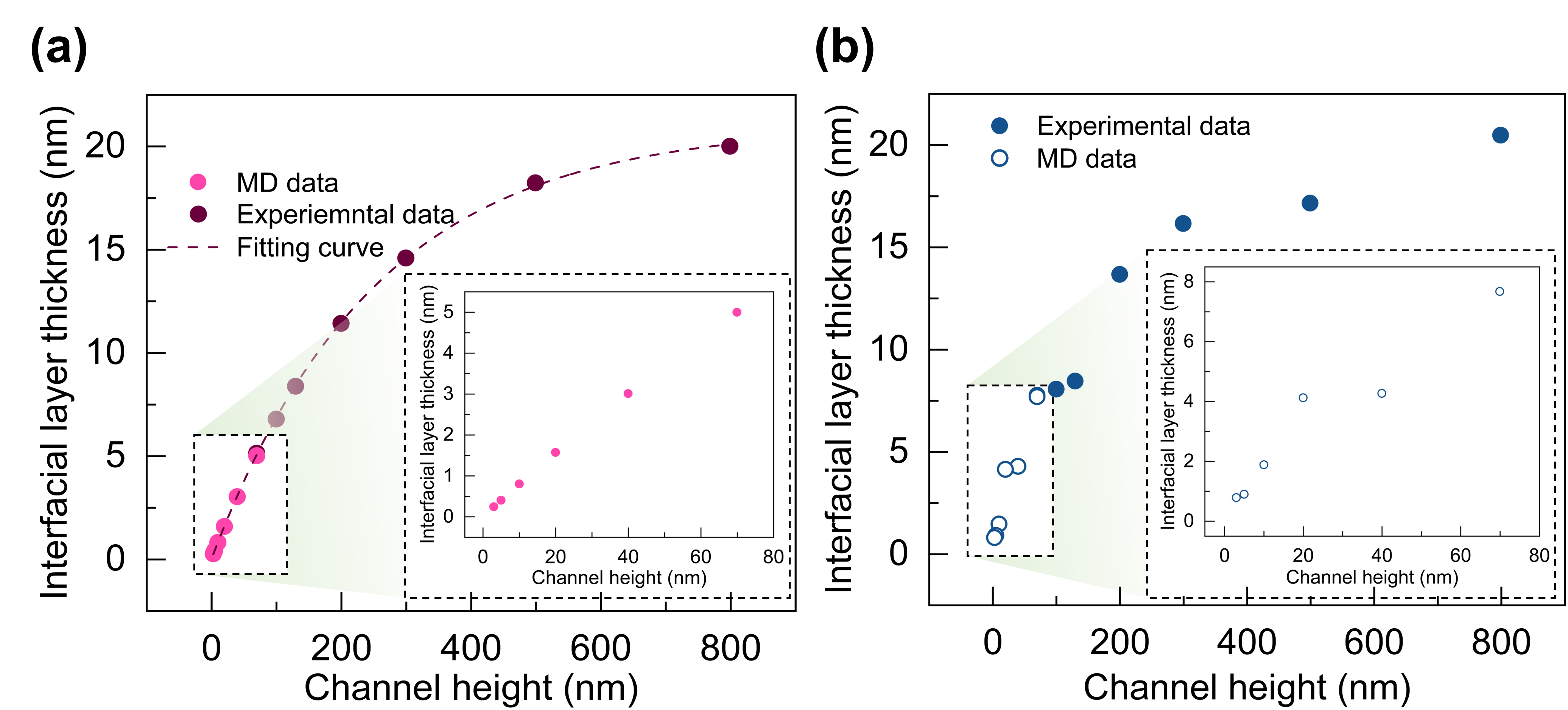


**Figure S14.** The relationship between the thickness of the interfacial layer and the channel height for **(a)** n-dodecane and **(b)** 3,3-dimethyldecane.

**

**

**Figure S15.** Comparison of interfacial layer viscosity of different fluids in a 10 nm nanochannel obtained from the predictive model and MD simulations.

# S8. Basic parameters in a molecular simulation study


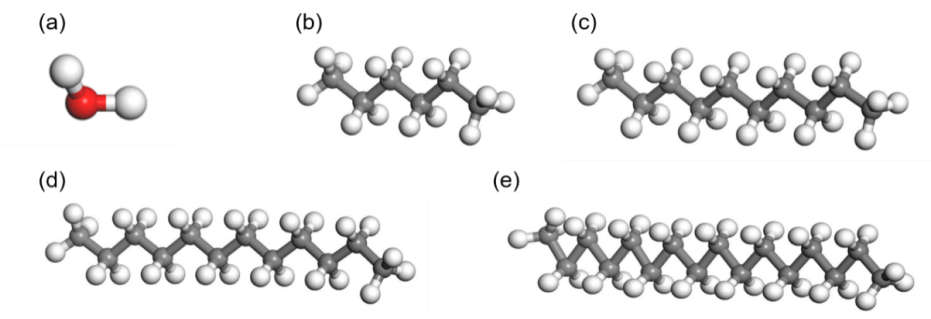


**Figure S16.** Molecular structure diagrams of fluids in the MD study. (a) Water molecule. (b) Hexane molecule. (c) Decane molecule. (d) Dodecane molecule. (e) Cetane molecule.

**
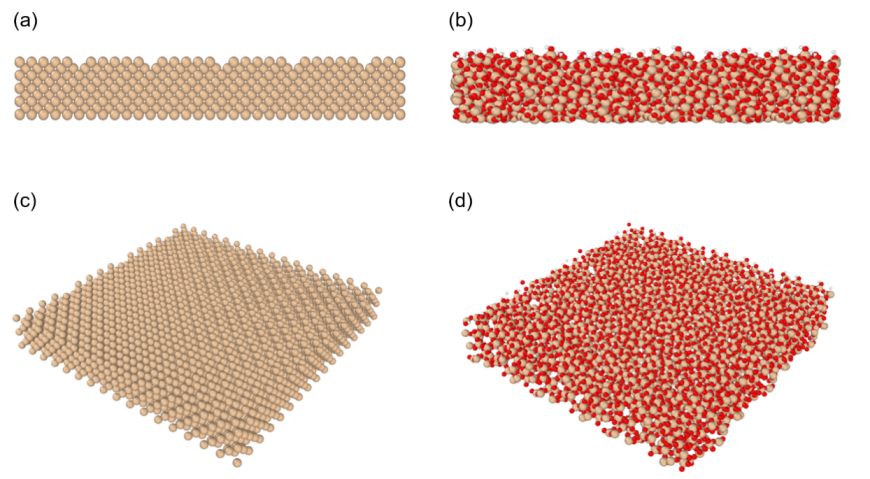
**

**Figure S17.** **Molecular arrangement of defective surface**. Molecular arrangement of **(a)** silicon wall and **(b)** silica. Oblique view of **(c)** silicon and **(d)** silica wall.


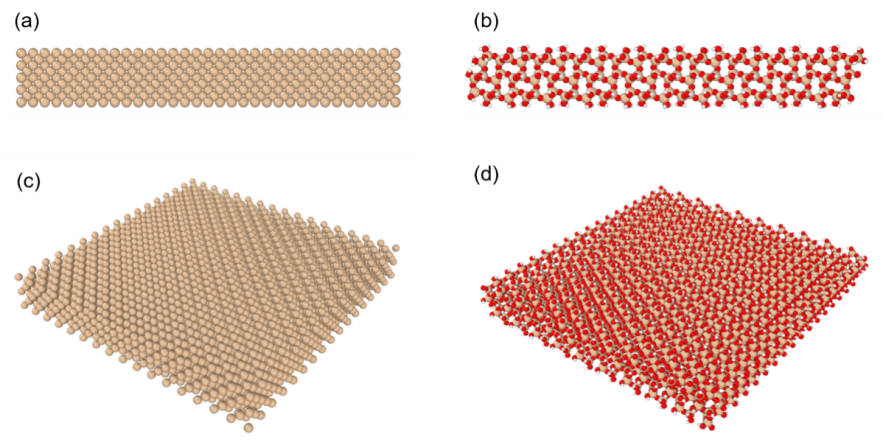


**Figure S18. Molecular arrangement of smooth surface**. Molecular arrangement of **(a)** silicon wall and **(b)** silica. Oblique view of **(c)** silicon and **(d)** silica wall.

**
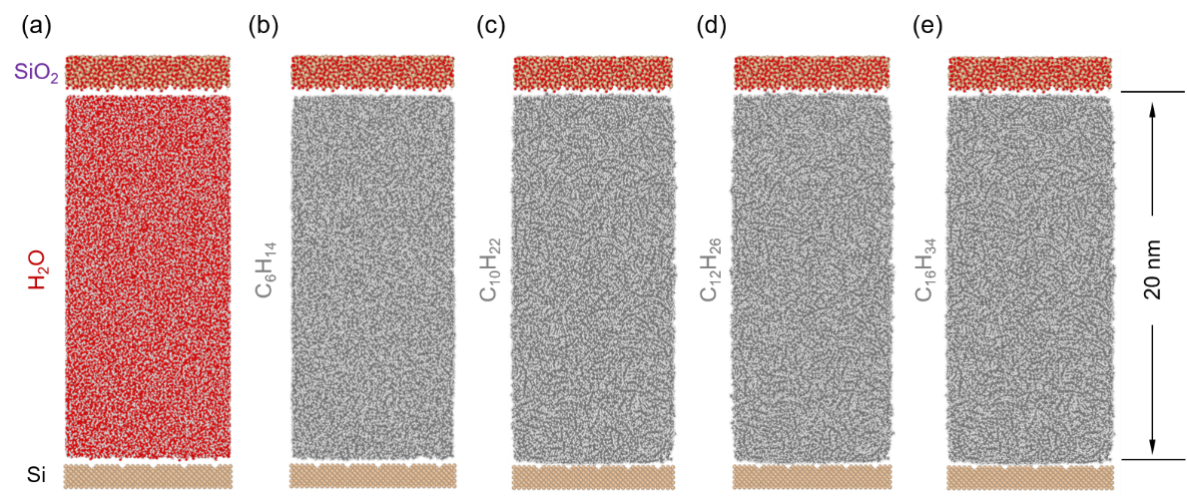
**

**Figure S19. The initial configuration status of fluid molecules in a nanochannel (Channel height: 20 nm)**. **(a)** Water molecule. **(b)** Hexane molecule. **(c)** Decane molecule. **(d)** Dodecane molecule. **(e)** Cetane molecule.

# S9. The calculation principle of intermolecular interaction forces

The potential energy function in molecular dynamics simulation consists of three parts: the non-bonding term, the bonding term, and the constraint term. The non-bonding term includes the Lennard-Jones term and the Coulomb term, namely, van der Waals forces and electrostatic forces. They are expressed as follows:

$E_{LJ}\left(r_{ij}\right)=4\varepsilon_{ij}\left[\left(\frac{\sigma_{ij}}{r_{ij}}\right)^{12}-\left(\frac{\sigma_{ij}}{r_{ij}}\right)^{6}\right]$ (S26)

$E_{C}\left(r_{ij}\right)=\frac{q_{i}q_{j}}{4\pi\varepsilon_{0}\varepsilon_{r}\varepsilon_{ij}}$ (S27)

For different atom types, the van der Waals forces are described using the Lorentz Berthelot mixing rule:

$\sigma_{ij}=\frac{1}{2}\left(\sigma_{ii}+\sigma_{jj}\right)$ (S28)

$\varepsilon_{ij}=\sqrt{\varepsilon_{ii}\times\varepsilon_{jj}}$ (S29)

where *E*_ij_ is the Lennard Jones potential between two atoms, *E*_c_(*r*_ij_) is the Coulombic interaction energy between two atoms, *σ* stands for the feature distance, namely, the distance between two molecules when the potential energy of molecular interaction is zero, *ε* represents the energy parameter, *r*_ij_ denotes the distance between the two atoms, *q* is the charge of atom. After configuring the initial state configuration of fluid molecules in the simulation channel, the energy of fluid molecules was released by the energy minimization method. Then, the isothermal isobaric ensemble (NPT) system was used to optimize the system. The system temperature was raised to 30 ^o^C, 40 ^o^C, and 50 ^o^C, and controlled by the Nose Hoover module^[7]^. The NPT optimization time is set to 20 ns, followed by an NPT simulation of 2 ns. The motion of atoms was described by the classical Newton equation and solved through the velocity-momentum algorithm.

# S10 The calculation principle of fluid viscosity and density

The calculation of fluid viscosity in a nanochannel is achieved by constructing a shear field combined with lateral linear momentum flux, and they are expressed as follows:

$j_{z}\left(p_{x}\right)=-\eta\frac{\partial v_{x}}{\partial z}$ (S30)

Thus, the *η* can be rewritten as:

$\eta=-\frac{\sum_{transfer} \left(P_{x,1}-P_{x,11}\right)}{2\text{Δ}tL_{x}L_{y}\frac{\partial v_{x}}{\partial z}}$ (S31)

where *j*_z_(*p*_x_) is the component in the *z* direction of the momentum *P_x_*, *η* represents the total viscosity of fluid molecules, *v_x_* stands for the horizontal velocity, *L*_x_, *L*_y_ are the

migration distance of fluid molecules in the *x* and *y* directions, $\left\langle\frac{\partial v_{x}}{\partial z} \right\rangle$ is the velocity gradient in the *z* direction. Construct a shear field in the nanochannel and calculate the momentum flux perpendicular to the shear direction, as shown in Figure S20. Divide the fluid into 20 units along the z-direction, and construct a velocity gradient by exchanging the momentum components of atoms in the x-direction between units 1, 20, and 11, thereby forming a shear field. Notably, this momentum exchange is a non-physical process. Consequently, the fluid viscosity was obtained by calculating the momentum exchange flux of physical properties in the z-direction. When the system reached the stable state, the mass and volume of fluid molecules along the z-direction in the nanochannel were counted, and the density distribution of fluid molecules in the simulation was calculated.


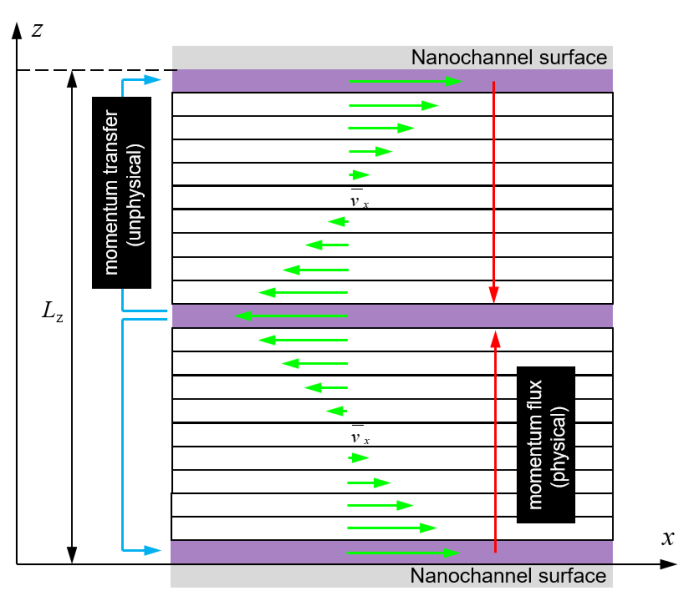


**Figure S20.** Schematic diagram of the momentum exchange method

# S11. The average interaction energy (AIE) of nanoconfined fluids

**Table S2** The AIE of nanoconfined fluids on rough channel surface from 30 to 50 ^o^C

| Temperature ^o^C | 30 | | | 40 | | | 50 | | |
| --- | --- | --- | --- | --- | --- | --- | --- | --- | --- |
| AIE (J/mol) | Liq-Liq | Liq-SiO_2_ | Liq-Si | Liq-Liq | Liq-SiO_2_ | Liq-Si | Liq-Liq | Liq-SiO_2_ | Liq-Si |
| H_2_O | 14158.84 | 1524.62 | 616.11 | 13682.45 | 1615.87 | 645.2 | 13356.31 | 1648.44 | 687.49 |
| C_6_H_14_ | 7955.17 | 2437.24 | 1183.76 | 7662.64 | 2285.15 | 1014.83 | 7504.62 | 2368.94 | 1230.42 |
| C_10_H_22_ | 9514.23 | 2684.57 | 1302.67 | 9369.66 | 2558.28 | 1208.64 | 9208.75 | 2504.33 | 1289.56 |
| C_12_H_26_ | 11006 | 2894.23 | 1584.68 | 9904.68 | 2861.47 | 1614.28 | 9839.62 | 2914.63 | 1628.32 |
| C_16_H_34_ | 14057.14 | 3525.6 | 1950.28 | 13785.76 | 3614.55 | 1996.47 | 13773.39 | 3657.83 | 2045.83 |
| 3,3-Dimethyltetradecane | 14985.14 | 3847.56 | 2284.32 | 14686.44 | 3954.87 | 2312.45 | 14530.28 | 3789.66 | 3896.11 |

**Table S3** The AIE of nanoconfined fluids on smooth channel surface at 30 ^o^C

| Fluid | H_2_O | C_6_H_14_ | C_10_H_22_ | C_12_H_26_ | C_16_H_34_ | 3,3-Dimethyltetradecane |
| --- | --- | --- | --- | --- | --- | --- |
| Liq-Liq (J/mol) | 13984.69 | 7715.72 | 9189.79 | 10580.07 | 12898.83 | 13687.52 |
| Liq-SiO_2_ (J/mol) | 1391.06 | 2026.81 | 1880.81 | 1883.35 | 1726.48 | 2024.71 |
| Liq-Si (J/mol) | 566.76 | 1045.38 | 1009.31 | 1184.47 | 1033.7 | 1195.3 |


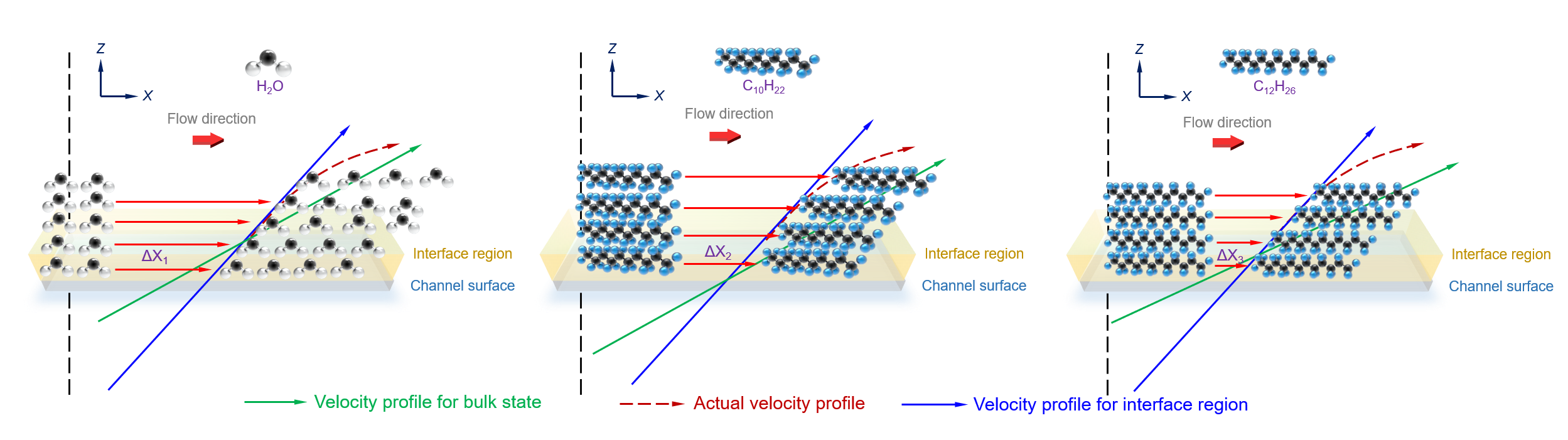


**Figure S21.** Schematic diagram of fluid molecules (H_2_O, C_10_H_22_, C_12_H_26_) moving on a wall.


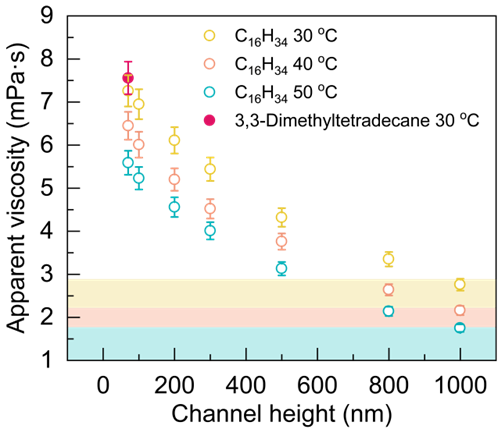


**Figure S22.** The apparent viscosity of C_16_H_34_ and 3,3-Dimethyltetradecane under different temperatures.


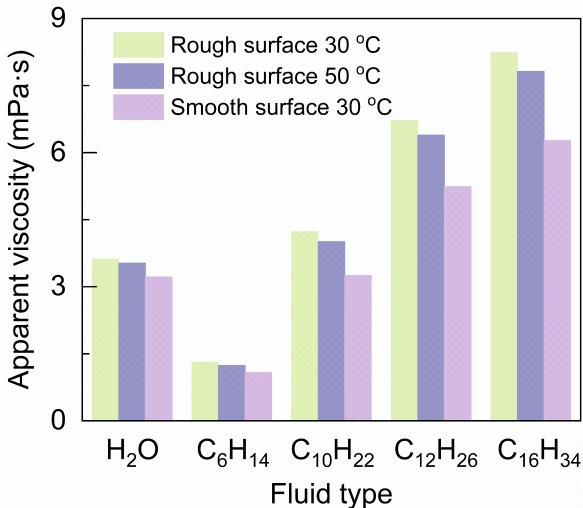


**Figure S23.** The effect of temperature and wall roughness on the apparent viscosity of nanoconfined fluids.


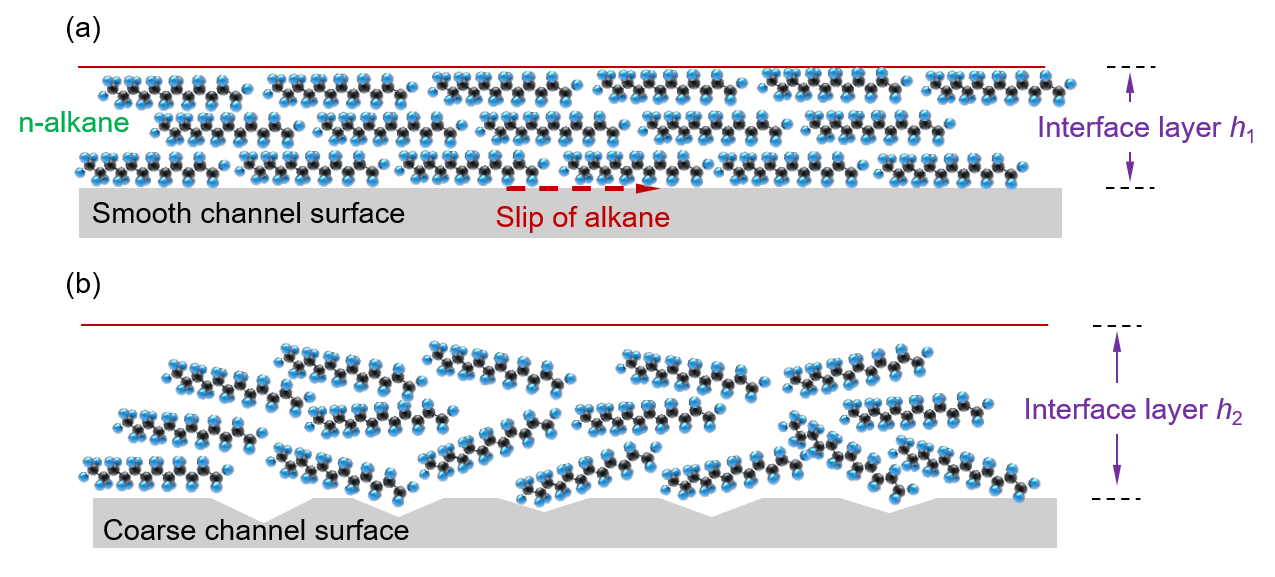


**Figure S24.** Molecular arrangement of n-alkane on smooth (a) and rough (b) channel surface.


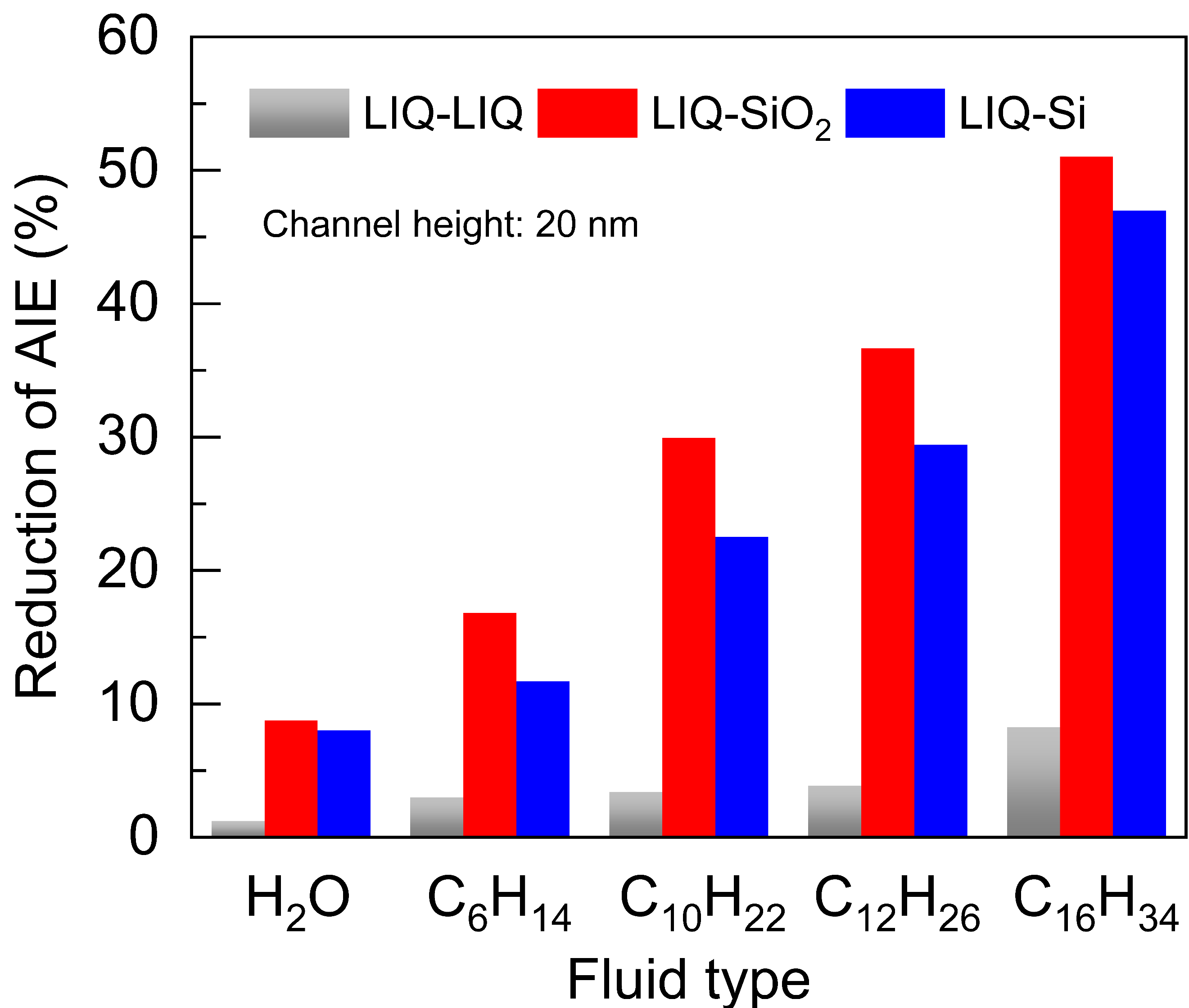


**Figure S25.** The reduction of AIE in the nanochannel with a height of 20 nm when the channel surface changed from rough to smooth.

# S12. Nanobubbles in the fluid imbibition process

Experimental findings of fluid imbibition presented a correlation between the appearance of nanobubbles and channel height, as well as fluid type (Figs. S26-S29). The influence of wall surface roughness became significant when the spatial scale was sufficiently small. In such a case, fluid tended to flow preferentially along areas with lower roughness, which thus produced a discontinuous flow. Moreover, fluid tended to flow along the channel surface to minimize the interfacial energy, forming the thin films along the edges. This was the so-called corner flow, and the effect became stronger for the thinner nanochannels^[8-10]^. The higher apparent viscosity of C_12_H_26_ in the interfacial layer could weaken the corner flow effect. Therefore, the discontinuous flow did not occur in the C_12_H_26_ imbibition, even though the channel height was 20 nm in this work (Figure S28). Although similar conclusions have been reported in other studies^[11,12]^, these works were primarily focused on confined spaces above 50 nm, and the fluid was mostly limited to water. These studies demonstrated that the relationship between the meniscus displacement (Δ*x*^2^) and time (Δ*t*) remained linear even in the presence of nanobubbles, and nanobubbles hardly impact the transport of nanoconfined fluids.

To evaluate the effect of nanobubbles, the comparative imbibition experiments with and without degassing were performed. The results showed no significant dependence on dissolved-gas removal, indicating that gas-related artifacts are unlikely to be the dominant factor (Figure S30a). Furthermore, the pressure difference across the chip at different injection rates was measured during the water injection (Non-degassed). The results showed that the pressure difference remained highly stable across all tested injection rates, without noticeable fluctuations (Figure S30b), indicating that bubble formation had no measurable influence during the injection process. The temperature testing results indicated that the temperature difference between the chip inlet and outlet was only 0.1 ^o^C at all tested temperatures (Figure S31), confirming the absence of interfacial thermal resistance throughout the experiments.


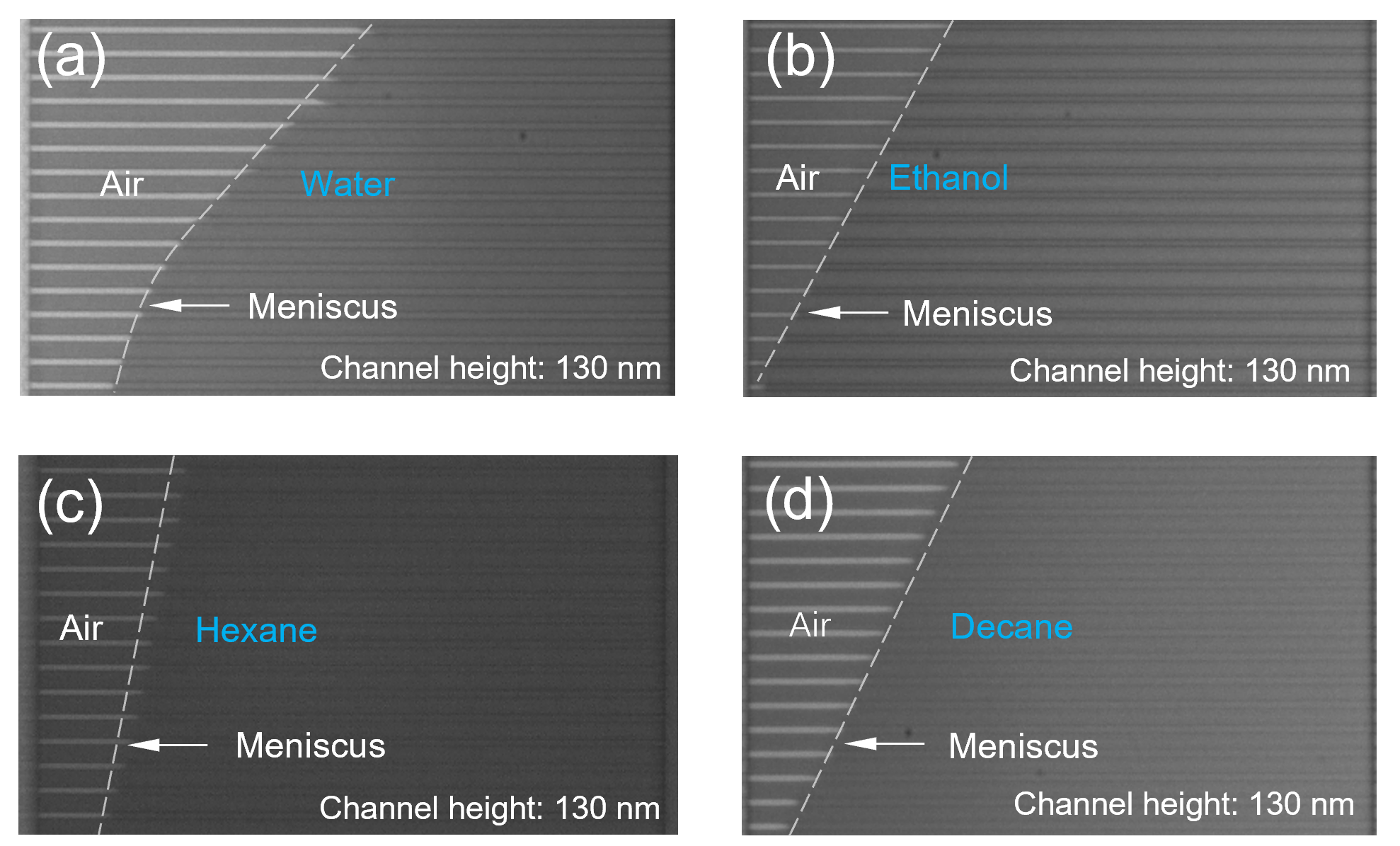


**Figure S26.** Fluids (water, ethanol, hexane, and decane) imbibition in the channel height of 130 nm under 30 ^o^C.


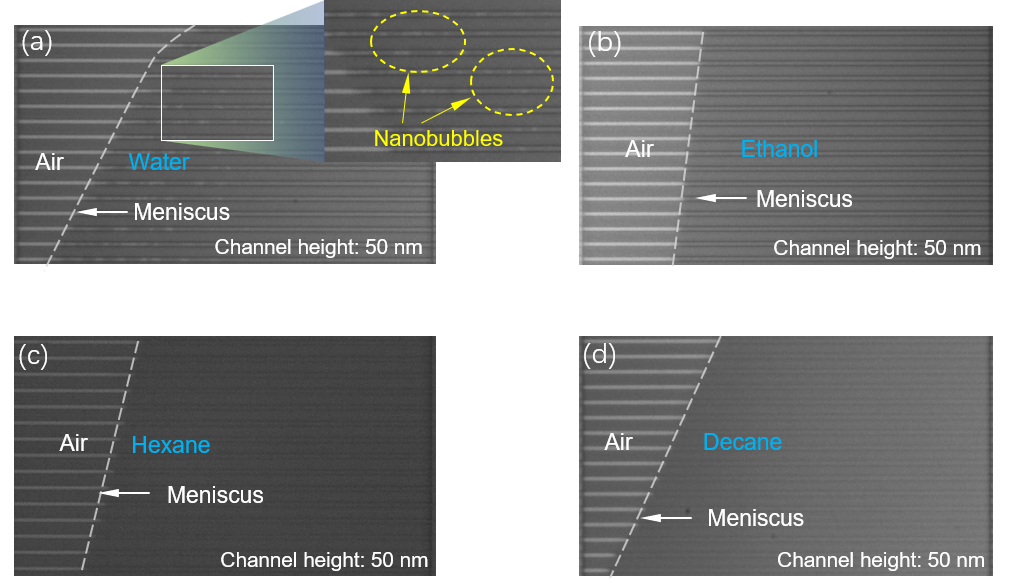


**Figure S27.** Fluids (water, ethanol, hexane, and decane) imbibition in the channel height of 50 nm under 30 ^o^C.


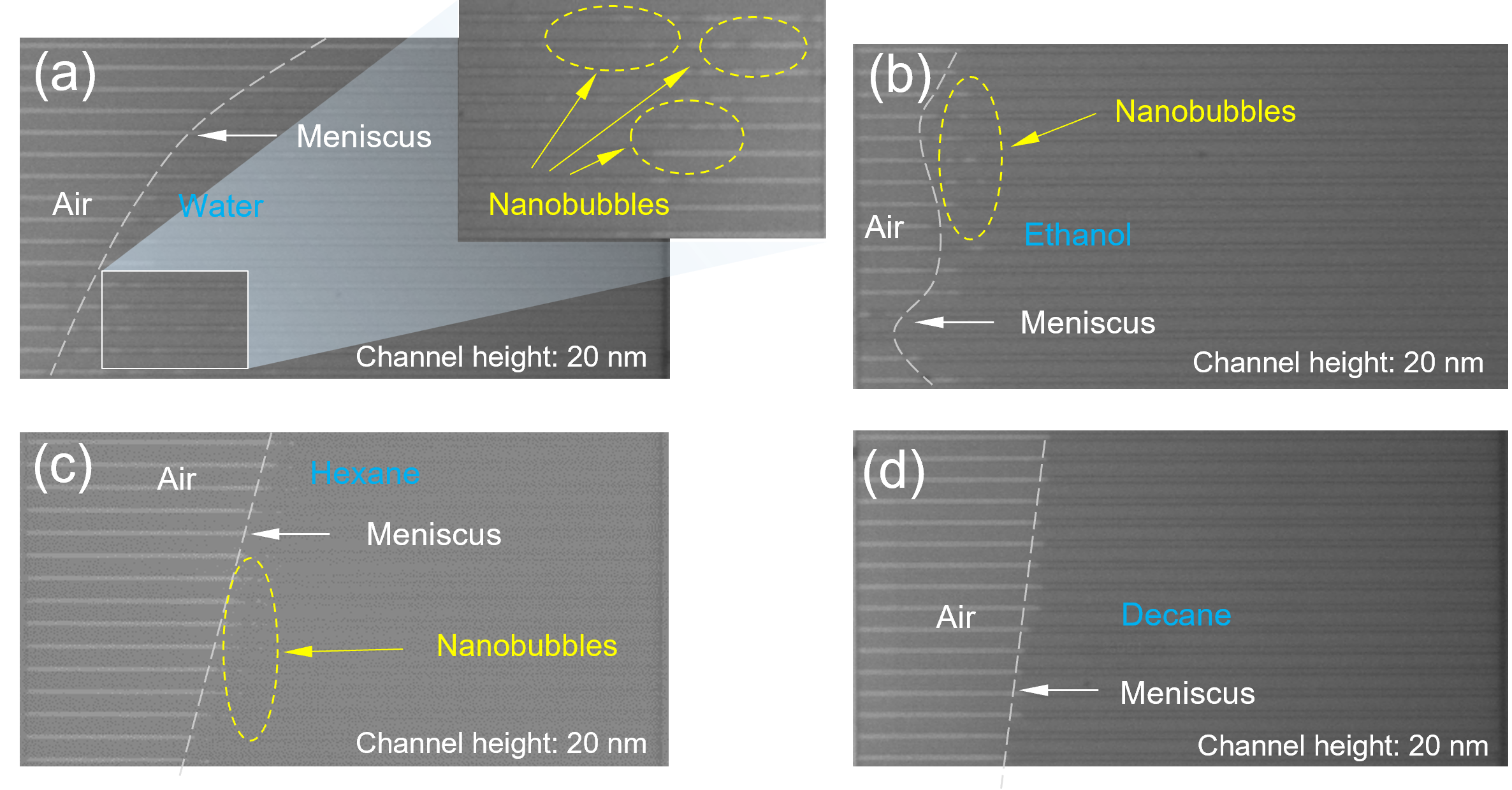


**Figure S28.** Fluids (water, ethanol, hexane, and decane) imbibition in the channel height of 20 nm under 30 ^o^C.


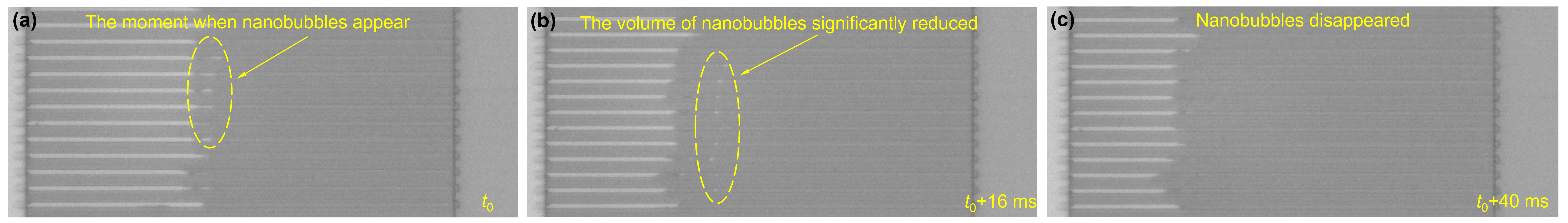


**Figure S29.** The generation and disappearance of nanobubbles


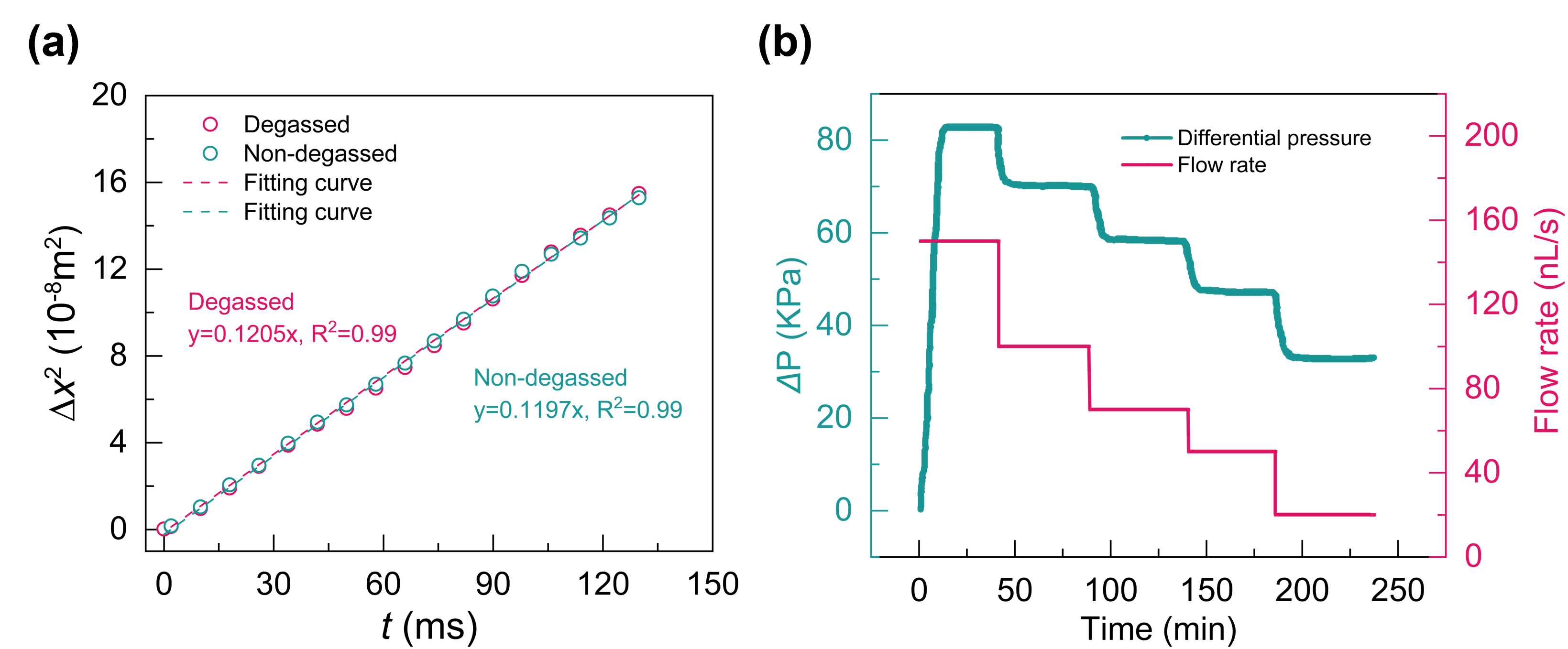


**Figure S30.** The **(a)** displacement-time curve during imbibition and **(b)** pressure difference curve during constant injection of water.





**Figure S31.** Temperature curves at the inlet and outlet of the chip during the water injection process under different temperature conditions.

# Reference

1. E. W. Washburn, *Phys. Rev.* **1921**, *17*, 273.
2. L. Li, Y. Kazoe, K. Mawatari, Y. Sugii, T. Kitamori, *J. Phys. Chem. Lett*. **2012**, *3*, 2447.
3. N. Ichikawa, K. Hosokawa, R. Maeda, *J. Colloid Interf. Sci*. **2004**, *280*, 155.
4. H. Lu, Y. Xu, C. Duan, P. Jiang, R. Xu, *Energy Fuels* **2022**, *36*, 5267.
5. M. Nazari, A. Davoodabadi, D. Huang, T. Luo, H. Ghasemi, *Nanoscale* **2020**, *12*, 14626.
6. M. Nazari, A. Masoudi, P. Jafari, P. Irajizad, V. Kashyap, H. Ghasemi, *Langmuir* **2019**, *35*, 78.
7. J. Li, J. D. Lee, *Acta Mech*. **2014**, *225*, 1223.
8. L. Shui, J. Eijkel, A. Vandenberg, *Sens. Actuators, B* **2007**, *121*, 263.
9. E. Kim, G. M. Whitesides, *J. Phys. Chem. B* **1997**, *101*, 855.
10. Eijkel J. et al. Strongly accelerated and humidity-independent drying of nanochannels induced by sharp corners. *Phys. Rev. Lett*. **95**, 256107 (2005).
11. M. Yang, B. Y. Cao, W. Wang, H. M. Yun, B. M. Chen, *Chem. Phy. Lett.* **2016**, *662*, 137.
12. A. Han, G. Mondin, N. G. Hegelbach, N. F. de Rooij, U. Staufer, *J. Colloid Interf. Sci.* **2006**, *293*, 151.

1. ⁎ *Corresponding author.*

   *E-mail address:* [*bwei@swpu.edu.cn*](mailto:bwei@swpu.edu.cn) *(B. Wei).* [↑](#footnote-ref-1)
